# Supplementary material for: Mortality and predictors among HIV-TB co-infected patients in Ethiopia: A systematic review and meta-analysis
Source: PLoS One. 2025 Jan 6;20(1):e0317048. doi: 10.1371/journal.pone.0317048 (PMC11703055; doi:10.1371/journal.pone.0317048)
Supplement: S2 Table — (DOCX) [file pone.0317048.s002.docx]

**Mortality and predictors among HIV-TB co-infected patients in Ethiopia: Systemic Review and Meta-analysis**

**S2 Table: Search Terms for PubMed, CINHAL, Global Index Medicus (GID).**

|  | Concept 1 | Concept 2 | Concept 3 | Concept 4 | |
| --- | --- | --- | --- | --- | --- |
| Free Text Word | Incidence | Mortality | “HIV/TB co-infected patients” | | Ethiopia |
| MeSH | "incidence"[MeSH Terms] "epidemiology"[Subheading] | - | - | | - |
| Related terms | Occurrence  Magnitude  Prevalence  Burden  Proportion  Epidemiology  Predictors  determinants  “Associated factors”  “Risk factors” | Death  Fatality  “survival status”  Survival  “survival rate” | “HIV-TB co-infected patients”  “HIV-TB co-infected cases”  “HIV-TB co-infection”  “HIV and TB co-infection”  “Tuberculosis/HIV co-infected patients”  “MTB/HIV co-infection”  Tuberculosis  TB  “Human immunodeficiency virus”  HIV/AIDS  TB/HIV  “HIV TB co-infected patients” | | “Addis Ababa”  “Dire Dawa”  “Benishangul-Gumuz”  Afar  Oromia  Somali  Tigray  Gambella  Harari  “Southern nations, nationalities and peoples’ region”  Amhara  “Sidama region”  SNNPR |

Pubmed search string

Date of search November 25/2023

From inception to November 25/2023

| Search in each key words and Mesh terms | **Pumed/medline database** |
| --- | --- |
| **#1** | ("Incidence"[MeSH Terms] OR "Epidemiology"[MeSH Subheading] OR "Incidence"[Title/Abstract] OR "Occurrence"[Title/Abstract] OR "Magnitude"[Title/Abstract] OR "Prevalence"[Title/Abstract] OR "Burden"[Title/Abstract] OR "Proportion"[Title/Abstract] OR "Epidemiology"[Title/Abstract] OR "Predictors"[Title/Abstract] OR "determinants"[Title/Abstract] OR "Associated factors"[Title/Abstract] OR "Risk factors"[Title/Abstract]) |
| **#2** | ("mortality"[Title/Abstract] OR "survival status"[Title/Abstract] OR "survival"[Title/Abstract] OR "survival rate"[Title/Abstract] OR "death"[Title/Abstract] OR "fatality"[Title/Abstract]) |
| **#3** | ("hiv tb co infected patients"[Title/Abstract] OR "hiv tb co infected patients"[Title/Abstract] OR "HIV-TB co-infected cases"[Title/Abstract] OR "HIV-TB co-infection"[Title/Abstract] OR "HIV and TB co-infection"[Title/Abstract] OR "Tuberculosis/HIV co-infected patients"[Title/Abstract] OR "hiv tb co infected patients"[Title/Abstract] OR "MTB/HIV co-infection"[Title/Abstract] OR "Tuberculosis"[Title/Abstract] OR "TB"[Title/Abstract] OR "Human immunodeficiency virus"[Title/Abstract] OR "hiv aids"[Title/Abstract] OR "tb hiv"[Title/Abstract]) |
| **#4** | ("Ethiopia"[Title/Abstract] OR "Addis Ababa"[Title/Abstract] OR "Dire Dawa"[Title/Abstract] OR "Benishangul-Gumuz"[Title/Abstract] OR "Afar"[Title/Abstract] OR "Oromia"[Title/Abstract] OR "Somali"[Title/Abstract] OR "Tigray"[Title/Abstract] OR "Gambella"[Title/Abstract] OR "Harari"[Title/Abstract] OR "southern nations nationalities and peoples region"[Title/Abstract] OR "SNNPR"[Title/Abstract] OR "Amhara"[Title/Abstract] OR "Sidama region"[Title/Abstract]) |
| **Final** | **(#1AND #2 AND #3 AND #4)** ("Incidence"[MeSH Terms] OR "Epidemiology"[MeSH Subheading] OR "Incidence"[Title/Abstract] OR "Occurrence"[Title/Abstract] OR "Magnitude"[Title/Abstract] OR "Prevalence"[Title/Abstract] OR "Burden"[Title/Abstract] OR "Proportion"[Title/Abstract] OR "Epidemiology"[Title/Abstract] OR "Predictors"[Title/Abstract] OR "determinants"[Title/Abstract] OR "Associated factors"[Title/Abstract] OR "Risk factors"[Title/Abstract]) AND ("mortality"[Title/Abstract] OR "survival status"[Title/Abstract] OR "survival"[Title/Abstract] OR "survival rate"[Title/Abstract] OR "death"[Title/Abstract] OR "fatality"[Title/Abstract]) AND ("hiv tb co infected patients"[Title/Abstract] OR "hiv tb co infected patients"[Title/Abstract] OR "HIV-TB co-infected cases"[Title/Abstract] OR "HIV-TB co-infection"[Title/Abstract] OR "HIV and TB co-infection"[Title/Abstract] OR "Tuberculosis/HIV co-infected patients"[Title/Abstract] OR "hiv tb co infected patients"[Title/Abstract] OR "MTB/HIV co-infection"[Title/Abstract] OR "Tuberculosis"[Title/Abstract] OR "TB"[Title/Abstract] OR "Human immunodeficiency virus"[Title/Abstract] OR "hiv aids"[Title/Abstract] OR "tb hiv"[Title/Abstract]) AND ("Ethiopia"[Title/Abstract] OR "Addis Ababa"[Title/Abstract] OR "Dire Dawa"[Title/Abstract] OR "Benishangul-Gumuz"[Title/Abstract] OR "Afar"[Title/Abstract] OR "Oromia"[Title/Abstract] OR "Somali"[Title/Abstract] OR "Tigray"[Title/Abstract] OR "Gambella"[Title/Abstract] OR "Harari"[Title/Abstract] OR "southern nations nationalities and peoples region"[Title/Abstract] OR "SNNPR"[Title/Abstract] OR "Amhara"[Title/Abstract] OR "Sidama region"[Title/Abstract]) **(n=623)** |
|  | **CINAHL (N=159)** |
| **#1** | =(Incidence+OR+Occurrence+OR+Magnitude+OR+Prevalence+OR+Burden+OR+Proportion+OR+Epidemiology+OR+Predictors+OR+determinants+OR+%26quot%3b Associated+factors%26quot%3b+OR+%26quot%3bRisk+factors%26quot%3b) |
| **#2** | +(mortality+OR+Death+OR+Fatality+OR+%26quot%3bsurvival+status%26quot%3b+OR+Survival+OR+%26quot%3bsurvival+rate%26quot%3b)+ |
| **#3** | +(%26quot%3bHIV%2fTB+co-infected+patients%26quot%3b+OR+%26quot%3bHIV-TB+co-infected+patients%26quot%3b+OR+%26quot%3bHIV-TB+co-infected+cases%26quot%3b+OR+%26quot%3bHIV-TB+co-infection%26quot%3b+OR+(%26quot%3bHIV+and+TB+co-infection%26quot%3b)+OR+%26quot%3bTuberculosis%2fHIV+co-infected+patients%26quot%3b+OR+%26quot%3bMTB%2fHIV+co-infection%26quot%3b+OR+Tuberculosis+OR+TB+OR+%26quot%3bHuman+immunodeficiency+virus%26quot%3b+OR+HIV%2fAIDS+OR+TB%2fHIV)+ |
| **#4** | +(Ethiopia+OR+%26quot%3bAddis+Ababa%26quot%3b+OR+%26quot%3bDire+Dawa%26quot%3b+OR+%26quot%3bBenishangul-Gumuz%26quot%3b+OR+Afar+OR+Oromia+OR+Somali+OR+Tigray+OR+Gambella+OR+Harari+OR+(%26quot%3bSouthern+nations%2c+nationalities++and+peoples%26%2339%3b+region%26quot%3b)+OR+Amhara)&type=1&searchMode=Standard&site=ehost-live&ssl |
| **Final** | **(#1AND#2AND#3AND#4)** (https://login.research4life.org/tacsgr1search_ebscohost_com/login.aspx?direct=true&db=cin20&bquery=(Incidence+OR+Occurrence+OR+Magnitude+OR+Prevalence+OR+Burden+OR+Proportion+OR+Epidemiology+OR+Predictors+OR+determinants+OR+%26quot%3bAssociated+factors%26quot%3b+OR+%26quot%3bRisk+factors%26quot%3b)+AND+(mortality+OR+Death+OR+Fatality+OR+%26quot%3bsurvival+status%26quot%3b+OR+Survival+OR+%26quot%3bsurvival+rate%26quot%3b)+AND+(%26quot%3bHIV%2fTB+co-infected+patients%26quot%3b+OR+%26quot%3bHIV-TB+co-infected+patients%26quot%3b+OR+%26quot%3bHIV-TB+co-infected+cases%26quot%3b+OR+%26quot%3bHIV-TB+co-infection%26quot%3b+OR+(%26quot%3bHIV+and+TB+co-infection%26quot%3b)+OR+%26quot%3bTuberculosis%2fHIV+co-infected+patients%26quot%3b+OR+%26quot%3bMTB%2fHIV+co-infection%26quot%3b+OR+Tuberculosis+OR+TB+OR+%26quot%3bHuman+immunodeficiency+virus%26quot%3b+OR+HIV%2fAIDS+OR+TB%2fHIV)+AND+(Ethiopia+OR+%26quot%3bAddis+Ababa%26quot%3b+OR+%26quot%3bDire+Dawa%26quot%3b+OR+%26quot%3bBenishangul-Gumuz%26quot%3b+OR+Afar+OR+Oromia+OR+Somali+OR+Tigray+OR+Gambella+OR+Harari+OR+(%26quot%3bSouthern+nations%2c+nationalities++and+peoples%26%2339%3b+region%26quot%3b)+OR+Amhara)&type=1&searchMode=Standard&site=ehost-live&ssl) **(N=159)** |
|  | **Global Index Medicus (N=25)** |
| #1 | tw:((tw:(tw:((tw:(incidence)) OR (tw:(occurrence)) OR (tw:(magnitude)) OR (tw:(prevalence)) OR (tw:(burden)) OR (tw:(proportion)) OR (tw:(epidemiology)) OR (tw:(predictors)) OR (tw:(determinants)) OR (tw:( “associated factors”)) OR (tw:( “risk factors”))))) |
| #2 | (tw:(tw:((tw:(mortality)) OR (tw:(death)) OR (tw:(fatality)) OR (tw:( “survival status”)) OR (tw:(survival))))) |
| #3 | (tw:(tw:( (tw:( “hiv/tb co-infected patients”)) OR (tw:( “hiv-tb co-infected patients”)) OR (tw:( “hiv-tb co-infected cases”)) OR (tw:( “hiv-tb co-infection”)) OR (tw:( “hiv and tb co-infection”)) OR (tw:( “tuberculosis/hiv co-infected patients”)) OR (tw:( “mtb/hiv co-infection”)) OR (tw:(tuberculosis)) OR (tw:(tb)) OR (tw:( “human immunodeficiency virus”)) OR (tw:(hiv/aids)) OR (tw:(tb/hiv)) OR (tw:( “hiv tb com-infected patients”))))) |
| #4 | **(**tw:(tw:((tw:(ethiopia)) OR (tw:( “addis ababa”)) OR (tw:( “dire dawa”)) OR (tw:( “benishangul-gumuz”)) OR (tw:(afar)) OR (tw:(oromia)) OR (tw:(somali)) OR (tw:(tigray)) OR (tw:(gambella)) OR (tw:(harari)) OR (tw:( “southern nations, nationalities AND peoples’ region”)) OR (tw:(amhara)) OR (tw:( “sidama region”)) OR (tw:(snnpr)))))) |
| Final | **(#1AND#2 AND#3 AND#4**) tw:((tw:(tw:((tw:(incidence)) OR (tw:(occurrence)) OR (tw:(magnitude)) OR (tw:(prevalence)) OR (tw:(burden)) OR (tw:(proportion)) OR (tw:(epidemiology)) OR (tw:(predictors)) OR (tw:(determinants)) OR (tw:( “associated factors”)) OR (tw:( “risk factors”))))) AND (tw:(tw:((tw:(mortality)) OR (tw:(death)) OR (tw:(fatality)) OR (tw:( “survival status”)) OR (tw:(survival))))) AND (tw:(tw:( (tw:( “hiv/tb co-infected patients”)) OR (tw:( “hiv-tb co-infected patients”)) OR (tw:( “hiv-tb co-infected cases”)) OR (tw:( “hiv-tb co-infection”)) OR (tw:( “hiv and tb co-infection”)) OR (tw:( “tuberculosis/hiv co-infected patients”)) OR (tw:( “mtb/hiv co-infection”)) OR (tw:(tuberculosis)) OR (tw:(tb)) OR (tw:( “human immunodeficiency virus”)) OR (tw:(hiv/aids)) OR (tw:(tb/hiv)) OR (tw:( “hiv tb co-infected patients”))))) AND (tw:(tw:((tw:(ethiopia)) OR (tw:( “addis ababa”)) OR (tw:( “dire dawa”)) OR (tw:( “benishangul-gumuz”)) OR (tw:(afar)) OR (tw:(oromia)) OR (tw:(somali)) OR (tw:(tigray)) OR (tw:(gambella)) OR (tw:(harari)) OR (tw:( “southern nations, nationalities and peoples’ region”)) OR (tw:(amhara)) OR (tw:( “sidama region”)) OR (tw:(snnpr)))))) **(n=25).** |
| **Free web search engines n=79** | |
| **Google Scholar** | (((Incidence OR Magnitude OR Predictors OR determinants OR “Associated factors”) AND (Mortality OR Death OR “survival status OR Survival OR "Survival rate ”) AND (“HIV/TB co-infected patients” OR “HIV-TB co-infection”OR “Tuberculosis/HIV co-infected patients” OR TB- “Human immunodeficiency virus ”) AND (Ethiopia OR “Addis Ababa”OR “Dire Dawa” OR “Benishangul-Gumuz” OR Afar OR Oromia OR Somali OR Tigray OR Gambella OR Harari OR “Southern nations nationalities and peoples’ region” OR Amhara OR “Sidama region” OR SNNPR )))) **(N=31)** |
| **Web of Sciences** | Incidence OR Magnitude OR Predictors OR determinants OR “Associated factors”AND (Mortality OR Death OR “AND (“HIV/TB co-infected patients” OR “HIV-TB co-infection”OR “Tuberculosis/HIV co-infected patients” OR TB- “Human immunodeficiency virus ”) AND (Ethiopia OR “Addis Ababa”OR “Dire Dawa” OR “Benishangul-Gumuz” OR Afar OR Oromia OR Somali OR Tigray OR Gambella OR Harari OR “Southern nations nationalities and peoples’ region” OR Amhara OR “Sidama region” OR SNNPR **(n=21)** |
| Google | Incidence OR Magnitude OR Predictors OR determinants OR “Associated factors”) AND (Mortality OR Death OR “survival status OR Survival) AND (“HIV/TB co-infected patients” OR “HIV-TB co-infection”OR “Tuberculosis/HIV co-infected patients” OR TB- “Human immunodeficiency virus ”) AND (Ethiopia OR “Addis Ababa”OR “Dire Dawa” OR “Benishangul-Gumuz” OR Afar OR Oromia OR Somali OR Tigray OR Gambella OR Harari OR “Southern nations nationalities and peoples’ region” OR Amhara OR “Sidama region” OR SNNPR **(N=27)** |
| **Total** | **886** |
