# Supplementary material for: Mortality and predictors among HIV-TB co-infected patients in Ethiopia: A systematic review and meta-analysis
Source: PLoS One. 2025 Jan 6;20(1):e0317048. doi: 10.1371/journal.pone.0317048 (PMC11703055; doi:10.1371/journal.pone.0317048)
Supplement: S3 Table — (DOCX) [file pone.0317048.s003.docx]

**S3 Table List of all identified studies in the literature search including those excluded studies from the analyses for the study of Mortality and predictors among HIV-TB co-infected patients in Ethiopia: A systematic review and meta-analysis.**

| **S.no** | **Author year** | **Eligibility status** | |
| --- | --- | --- | --- |
|  |  | **Included** | **Excluded studies and their reason.** |
|  | Mega et al., 2020, |  | Title and abstract |
|  | Meressa et al., 2015a, , |  | Title and abstract |
|  | Meressa et al., 2015b, |  | Title and and abstract |
|  | Bendayan et al., 2010, |  | Title and outcone , setting |
|  | Tessema and Alemu, 2021, |  | Title and abstract |
|  | Dawit, et al, 2021 | Included |  |
|  | Biressaw et al., 2013, |  | Title and abstract |
|  | Legesse and Reta, 2019, |  | Title and abstract |
|  | Arage et al., 2014, |  | Title and abstract |
|  | Murray et al., 1978, |  | Title |
|  | Offei-ansah, 2000, |  | Title, abstract, and study area |
|  | Fenta et al., 2020, |  | Title and abstract |
|  | Deressa et al., 2018, |  | Title and abstract |
|  | Jerene et al., 2006, |  | Title and abstract |
|  | Nega et al., 2020, |  | Title and abstract |
|  | Getawa et al., 2021, |  | Title and abstract |
|  | Hassen Ali et al., 2013, |  | Title and abstract |
|  | Getahun et al., 2023, |  | Title and abstract |
|  | Tolosie and Sharma, 2014, |  | Title and abstract |
|  | Weldearegawi et al., 2014, |  | Title and abstract |
|  | Jibril et al., 2018, |  | Title and abstract |
|  | Verguet et al., 2016, |  | Title and abstract |
|  | Gemechu, et al,2022 | Included |  |
|  | Chanie, et al, 2021 | Included |  |
|  | Alemayehu and Eyuel, 2011, |  | Title and abstract |
|  | Bizuayehu et al., 2015 |  | Title and abstract |
|  | Abossie and Yohanes, 2017, |  | Title and abstract |
|  | Mengesha and Ahmed, 2020, |  | Title and abstract |
|  | Demissie Gizaw et al., 2015, |  | Title and abstract |
|  | Wondifraw Baynes et al., 2017, |  | Title and abstract |
|  | Tesfaye et al., 2022, |  | Title and abstract |
|  | Diriba and Awulachew, 2022, |  | Title and abstract |
|  | Alebel et al., 2023, |  | Title and abstract |
|  | Bein and Coker-Farrell, 2020, |  | Title and abstract |
|  | Greig et al., 2012, |  | Title and abstract |
|  | Misganaw et al., 2013 |  | Title and abstract |
|  | Atallel, et al 2018 | Included |  |
|  | , Dejen et al., 2021, |  | Title and abstract |
|  | Tilahun et al., 2023, |  | Title and abstract |
|  | Rudolf, 2014, |  | Title and abstract |
|  | Sagbakken et al., 2008, |  | Title and abstract |
|  | Telele et al., 2018, |  | Title and abstract |
|  | Buta et al., 2015, |  | Title and abstract |
|  | Umeta et al., 2022, |  | Title and abstract |
|  | Mohamed, 2020, |  | Title and abstract |
|  | Genetu et al., 2017, |  | Title and abstract |
|  | Nega et al., 2019, |  | Title and abstract |
|  | Fantahun and Degu, 2004, |  | Title and abstract |
|  | Getaneh et al., 2023, |  | Title and abstract |
|  | Deribew et al., 2019, |  | Title and abstract |
|  | Ghislain et al., 2021b |  | Title and abstract |
|  | Ghislain et al., 2021a |  | Title |
|  | Umeta et al., 2021, |  | Title and abstract |
|  | Meles et al, ., 2023, |  | Title and abstract |
|  | Jima et al., 2013, |  | Title and abstract |
|  | Melaku et al., 2014, |  | Title and abstract |
|  | Ayele and Amogne, 2021 |  | Title and abstract |
|  | Dessalegn Mekonnen, 2020, |  | Title |
|  | Tesfaye et al., 2023, |  | Title and abstract |
|  | Leyh-Bannurah et al., 2014, et al., 2022, |  | Title, abstract , and outcome |
|  | Damtie et al., 2013m |  | Title and abstract |
|  | Welekidan et al., 2020, |  | Title and abstract |
|  | Ramos et al., 2010a, |  | Title and abstract |
|  | Ramos et al., 2010b, |  | Title and abstract |
|  | Hailu et al., 2014, |  | Title and abstract, outcome |
|  | Manaye et al., 2020 |  | Title and abstract |
|  | , Badacho et al., 2023, |  | Title and abstract |
|  | Beyene and Moss, 1991, |  | Title and abstract |
|  | Lenjiso et al., 2019, |  | Title and abstract |
|  | Hurissa et al., 2010, |  | Title and abstract |
|  | Negussie, et al,2021 | Included |  |
|  | Sultan et al., 2021, |  | Title and abstract |
|  | Geteneh et al., 2021, |  | Title and abstract |
|  | Abraha et al., 2021, |  | Title and abstract |
|  | Lester, 1992 |  | Title and abstract |
|  | Negera and Mega, 2019 |  | Title and abstract |
|  | Ayele et al., 2017, |  | Title and abstract |
|  | Shimeles and LulsegJed, 1994, |  | Title and abstract |
|  | Abdela et al., 2020, |  | Title and abstract |
|  | Korma et al., 2015, |  | Title and abstract |
|  | Batu et al., 2023, |  | Title and abstract |
|  | Tesfu et al., 2022, |  | Title and abstract |
|  | Damtie et al., 2013, |  | Title and abstract |
|  | Ayal and Berha, 20232020) |  | Title and abstract |
|  | Kumela et al., 2015 |  | Title and abstract |
|  | Ritmeijer et al., 2006, |  | Title and abstract |
|  | Fufa et al., 2023, |  | Title and abstract |
|  | Tadesse et al., 2016, |  | Title and abstract |
|  | Haji et al., 2014, |  | Title and abstract |
|  | ter Horst et al., 2008, |  | Title and abstract |
|  | Belay et al., 2021, |  | Title and abstract |
|  | Breman et al., 2004, |  | Title and abstract |
|  | Melaku and Zeleke, 2014, |  | Title and abstract |
|  | Accorsi et al., 2010, |  | Title, abstract, and study area |
|  | Pathak et al., 2021, |  | Title, and abstract |
|  | Ademas et al., 2021, |  | Title and abstract |
|  | Shimelis et al., 2016, |  | Title and abstract |
|  | Yimer et al., 2016, |  | Title and abstract |
|  | Ayalew et al., 2020, |  | Title |
|  | Tedla et al., 2020, |  | Title and abstract |
|  | Mesfin et al., 2009, |  | Title and abstract |
|  | Asres et al., 2019, |  | Title and abstract |
|  | Asres et al., 2018, |  | Title |
|  | Bohn et al., 2016, |  | Title and abstract |
|  | Mekonnen et al., 2002, |  | Title and abstract |
|  | Abdurehman and Enquoselassie, 2001, |  | Title and abstract |
|  | Edessa and Likisa, 2015, |  | Title and abstract |
|  | Balcha et al., 2014, |  | Title and abstract |
|  | Kibret et al., 2013, |  | Title and abstract |
|  | Gessesse et al., 2015, |  | Title and abstract |
|  | Alula, et al, 2017 | Included |  |
|  | Nugus and Irena,2020 |  | Title and abstract |
|  | Bokore et al., 2018, |  | Title and abstract |
|  | Nigusie et al., 2021, |  | Title and abstract |
|  | Zenu et al., 2021 |  | Title and abstract |
|  | Alem et al., 2022, |  | Title and abstract |
|  | Yibeltal et al., 2020, |  | Title and abstract |
|  | Kefeni et al., 2023, |  | Title and abstract |
|  | Hirpa et al., 2013 |  | Title and abstract |
|  | Tewachew et al., 2021, |  | Title and abstract |
|  | Wakeyo et al., 2020, |  | Title |
|  | Sintayehu et al., 2022, |  | Title |
|  | Alemu and Sebastián, 2010, |  | Title and abstract |
|  | Shargie and Lindtjørn, 2007, |  | Title, abstract , and study area |
|  | Tesema et al., 2020, |  | Title and abstract |
|  | Negessie et al., 2019, |  | Title and abstract |
|  | Gebrezgabher et al., 2017a, |  | Title and abstract |
|  | Gebrezgabher et al., 2017b, |  | Title and abstract |
|  | Abongomera et al., 2017, |  | Title and abstract |
|  | Alene et al., 2020, |  | Title and abstract |
|  | Hall et al., 2011, |  | Title and abstract |
|  | Workneh et al., 2016, |  | Title and abstract |
|  | Feleke et al., 2007, |  | Title and abstract |
|  | Herlihy et al., 2016 |  | Title and abstract |
|  | Quigley et al., 1999, |  | Title and abstract |
|  | Yimer et al., 2005, , |  | Title and abstract |
|  | Chesdachai et al., 2020, |  | Title |
|  | Kebede et al., 2021, |  | Title and abstract |
|  | Jerene and Lindtjørn, 2005 |  | Title and abstract |
|  | Tufa et al., 2023, |  | Title |
|  | Silva, 2018, |  | Title and abstract |
|  | Adamu et al., 2014, |  | Title and abstract |
|  | Getnet et al., 2017, |  | Title and abstract |
|  | Atey et al., 2020, |  | Title and abstract |
|  | Aderie et al., 2017, |  | Title and abstract |
|  | Eyob et al., 2004, |  | Title and abstract |
|  | Sosna et al., 1999 |  | Title and abstract |
|  | Shaweno, et al, 2012 | Included |  |
|  | Kemal et al., 2020, |  | Title and abstract |
|  | Tekola et al., 2008a, |  | Title and abstract |
|  | Tekola et al., 2008b |  | Title and abstract |
|  | , Girum et al., 2020, |  | Title and abstract |
|  | Sime et al., 2022, |  | Title and abstract |
|  | Tiruneh and Deyas, 2020, |  | Title and abstract |
|  | Assefa et al., 2014, |  | Title and abstract |
|  | Amare et al., 2023, |  | Title and abstract |
|  | Assebe et al., 2015, |  | Title and abstract |
|  | Beshaw et al., 2021, |  | Title and abstract |
|  | Chandramohan et al., 2001, |  | Title and abstract |
|  | Wariyo et al., 2022, |  | Title and abstract |
|  | Tesfaye et al., 2021 |  | Title and abstract |
|  | Molla et al., 2022, |  | Title and abstract |
|  | Alebel et al., 2022, |  | Title and abstract |
|  | Alebel et al., 2018, |  | Title and abstract |
|  | Manyazewal et al., 2020, |  | Title |
|  | Weldearegawi et al., 2013 |  | Title and abstract |
|  | Mascie-Taylor, 1992, |  | Title and abstract |
|  | Ding et al., 2022, |  | Title |
|  | Nigatu and Abraha, 2010a) |  | Title and abstract |
|  | , Nigatu and Abraha, 2010b, |  | Title and abstract |
|  | Burusie et al., 2023, |  | Title and abstract |
|  | Dagne et al., 2021, |  | Title and abstract |
|  | Gebreyes, 2023 | Included |  |
|  | Girum et al., 2018, |  | Title and abstract |
|  | Kebede et al., 2020, |  | Title and abstract |
|  | Tensou et al., 2010, |  | Title and abstract |
|  | Tadesse and Tadesse, 2012, |  | Title and abstract |
|  | Demissie et al., 2020, |  | Title and abstract |
|  | Sisay et al., 2018, |  | Title and abstract |
|  | Kahase et al., 2020, |  | Title |
|  | Bayissa et al., 2021, |  | Title and abstract |
|  | Tesfaye et al., 2020, |  | Title and abstract |
|  | Sidamo et al., 2021 |  | Title and abstract |
|  | Agegnehu and Alem, 2021, |  | Title and abstract |
|  | Abebe et al., 2010 |  | Title and abstract |
|  | Wondimu et al., 2020, |  | Title |
|  | Walle et al., 2022, |  | Title and abstract |
|  | Kaso et al., 2022, |  | Title and abstract |
|  | Seid and Metaferia, 2018, |  | Title and abstract |
|  | Adenager et al., 2017, |  | Title and abstract |
|  | Tedla et al., 2020b, |  | Title and abstract |
|  | Habte et al., 2020, |  | Title and abstract |
|  | Nezenega et al., 2020, |  | Title and abstract |
|  | Desalegn et al., 2022, |  | Title and abstract |
|  | Feyissa et al., 2020, |  | Title and abstract |
|  | Mosisa et al., 2020, |  | Title and abstract |
|  | Hamusse et al., 2014, |  | Title and abstract |
|  | Mengesha et al., 2021, |  | Title and abstract |
|  | Woldesemayat et al., 2015, |  | Title and abstract |
|  | Seid et al., 2022, |  | Title and abstract |
|  | W/Gebreal, et al, 2018 | Included |  |
|  | Girum et al., 2018b |  | Title and abstract |
|  | Tesfaye and Bune, 2014, |  | Title and abstract |
|  | Lobie et al., 2020, |  | Title and abstract |
|  | Melaku et al., 2020, |  | Title and abstract |
|  | Melaku et al., 2005 |  | Title and abstract |
|  | , Assefa et al., 2016, |  | Title and abstract |
|  | Hodes and Kloos, 1988, |  | Title and abstract |
|  | Alema et al., 2019 |  | Title and abstract |
|  | Verguet and Jamison, 2017, |  | Title |
|  | Reniers and Tesfai, 2009, |  | Title and abstract |
|  | Arja et al., 2022, |  | Title and abstract |
|  | W/Gebreal, et al, 2018 |  | Title and abstract |
|  | Damtie et al., 2021, |  | Title and abstract |
|  | Ayelign et al., 2021, |  | Title and abstract |
|  | Gedefie et al., 2023, |  | Title and abstract |
|  | Yami et al., 2011, |  | Title and abstract |
|  | Desalegn et al., 2016, |  | Title and abstract |
|  | Gedefie et al., 2021, |  | Title and abstract |
|  | Mulu et al., 2013, |  | Title and abstract |
|  | Gashaw et al., 2019 |  | Title and abstract |
|  | Wubshet et al., 2012, |  | Title and abstract |
|  | Milkias et al., 2023, |  | Title and abstract |
|  | Akalu et al., 2022, |  | Title and abstract |
|  | Jerene et al., 2022, |  | Title and abstract |
|  | Zohar et al., 2014, |  | Title, abstract and study area |
|  | Nigussie et al., 2021 |  | Title and abstract |
|  | Adane et al., 2012, |  | Title and abstract |
|  | Negese et al., 2012, |  | Title and abstract |
|  | Mishore et al., 2020 |  | Title |
|  | Weldearegawi et al., 2015 |  | Title and abstract |
|  | Solomon et al., 2021 |  | Title and abstract |
|  | Mengistu et al., 2015, |  | Title and abstract |
|  | Huruy et al., 2010, |  | Title and abstract |
|  | Tilahun et al., 2022, |  | Title and abstract |
|  | Hailu and Wasihun, 2021, |  | Title and abstract |
|  | Kiros et al., 2022, |  | Title and abstract |
|  | Omer and Mariam, 2008, |  | Title and abstract |
|  | Getaneh et al., 2022, |  | Title and abstract |
|  | Desta et al., 2021, |  | Title and abstract |
|  | Habtamu, et al, 2021 | Included |  |
|  | Feleke et al., 2020, |  | Title and abstract |
|  | Alemu et al., 2020b, |  | Title and abstract |
|  | Sherfa et al., 2021, |  | Title and abstract |
|  | Tigabu et al., 2022 |  | Title and abstract |
|  | Techane et al., 2020, |  | Title and abstract |
|  | Bimer et al., 2021, |  | Title and abstract |
|  | Wondifraw et al., 2022b, |  | Title and abstract |
|  | Alemu et al., 2020a, |  | Title and abstract |
|  | Arefaine et al., 2020, |  | Title and abstract |
|  | Mulugeta et al., 2021, | Included | Title and abstract |
|  | Abrha, et al, 2015 |  | Title and abstract |
|  | Azmeraw et al., 2022 |  | Title and abstract |
|  | Shumet and Kebede, 2023, |  | Title and abstract |
|  | Shiferaw et al., 2022, |  | Title and abstract |
|  | Menshw et al., 2021, |  | Title and abstract |
|  | Birhanu et al., 2020,. |  | Title and abstract |
|  | Geremew et al., 2023, |  | Title and abstract |
|  | Kassa et al., 2019, |  | Title and abstract |
|  | Birhan, et al, 2021 |  | Title and abstract |
|  | Gezae et al., 2019, |  | Title and abstract |
|  | Dawit et al., 2021, |  | Title and abstract |
|  | Refera, et al, 2013 | Included |  |
|  | Dagnaw Tegegne et al., 2022, |  | Title and abstract |
|  | Woldegeorgis et al., 2022 |  | Title and abstract |
|  | Meseret et al., 2017 |  | Title and abstract |
|  | Gebremeskel et al., 2021, |  | Title and abstract |
|  | Gedfew et al., 2020, |  | Title and abstract |
|  | Addis Alene et al., 2013, |  | Title and abstract |
|  | Wondifraw et al., 2022a, |  | Title and abstract |
|  | Tekese et al., 2023, |  | Title and abstract |
|  | Azanaw et al., 2021 |  | Title and abstract |
|  | Ayalaw et al., 2015 |  | Title and abstract |
|  | Temesgen et al., 2019, |  | Title and abstract |
|  | Beshir et al., 2019, |  | Title and abstract |
|  | Masino-Tessu et al., 2019, |  | Title |
|  | Aemro et al., 2020, |  | Title and abstract |
|  | Zakaria et al., 2022, |  | Title and abstract |
|  | Menza, 2022, |  | Title and abstract |
|  | Kerebeh et al., 2022, |  | Title and abstract |
|  | Gezae, et al, 2019 | Included |  |
|  | Wondwossen et al., 2016b, |  | Title and abstract |
|  | Wondwossen et al., 2016a, |  | Title and abstract |
|  | Melkamu et al., 2020, |  | Title and abstract |
|  | Barata et al., 2023, |  | Title and abstract |
|  | Teshale et al., 2021, |  | Title and abstract |
|  | Dagnaw et al., 2023, |  | Title and abstract |
|  | Mengesha et al., 2022, |  | Title and abstract |
|  | Lelisho, et al, 2022 | Included |  |
|  | Sibhat et al., 2023, |  | Title and abstract |
|  | Kassa et al., 2012, |  | Title and abstract |
|  | Aynalem et al., 2023, |  | Title and abstract |
|  | Endalamaw et al., 2018, |  | Title and abstract |
|  | Andarge et al., 2022, , |  | Title and abstract |
|  | Rudolf et al., 2021, |  | Title and abstract |
|  | Mor et al., 2013, |  | Title and abstract |
|  | Moges et al., 2006, |  | Title and abstract |
|  | Treister-Goltzman et al., 2021, |  | Title and abstract |
|  | Abongomera et al., 2018 |  | Title and abstract |
|  | Qazi and Muhe, 2006 |  | Title |
|  | Mhimbira et al., 2017, |  | Title and abstract |
|  | Gedle et al., 2017, |  | Title and abstract |
|  | Assefa et al., 2022 |  | Title and abstract |
|  | Mulatu et al., 2023, |  | Title and abstract |
|  | Ayana et al., 2021, |  | Title and abstract |
|  | Anjullo et al., 2023 |  | Title and abstract |
|  | Dessu et al., 2020, |  | Title and abstract |
|  | Demessie et al., 2014, |  | Title and abstract |
|  | Ramlan et al., 2020, , |  | Title and abstract |
|  | Abtew et al., 2016, |  | Title and abstract |
|  | Gesesew et al., 2018, |  | Title and abstract |
|  | Belay et al., 2019, |  | Title and abstract |
|  | Araya et al., 2004, |  | Title and abstract |
|  | Sifr et al., 2021, |  | Title and abstract |
|  | Tarekegn et al., 2019, |  | Title and abstract |
|  | Gebreegziabhier Kindaya and Kassaw Demoze, 2020, |  | Title and abstract |
|  | Ritmeijer et al., 2011, |  | Title and abstract |
|  | Diro et al., 2018, |  | Title and abstract |
|  | Sileshi, et al, 2013 | Included |  |
|  | Dangisso et al., 2018, |  | Title and abstract |
|  | Gemechu et al., 2023, |  | Title and abstract |
|  | Bantie et al., 2022, |  | Title and abstract |
|  | Dangisso et al., 2015, |  | Title and abstract |
|  | Tola et al., 2020, |  | Title and abstract |
|  | Mamo et al., 2023, |  | Title and abstract |
|  | Torres-Anjel, 1992, |  | Title, abstract, and setting |
|  | Tesfaye et al., 2020a, |  | Title and abstract |
|  | Tamir et al., 2019 |  | Title and abstract |
|  | Bayleyegn et al., 2021, |  | Title and abstract |
|  | Kiros et al., 2020 |  | Title and abstract |
|  | Ataro et al., 2019, |  | Title |
|  | Fetensa et al., 2022, |  | Title and abstract |
|  | Alema et al., 2022, |  | Title and abstract |
|  | Soboka et al., 2020, |  | Title and abstract |
|  | Kebede et al., 2018, |  | Title and abstract |
|  | Gudina et al., 2017, |  | Title and abstract |
|  | Alemayehu et al., 2020, |  | Title and abstract |
|  | Lukas et al., 2021 |  | Title and abstract |
|  | Diriba et al., 2021b, |  | Title and abstract |
|  | Demissie and Belayneh, 2021, , |  | Title and abstract |
|  | Kassa et al., 2022, |  | Title and abstract |
|  | Weldegebreal et al., 2018, |  | Title and abstract |
|  | Weldearegawi et al., 2020 |  | Title and abstract |
|  | Wonde et al., 2019, |  | Title |
|  | Tadesse et al., 2023, |  | Title and abstract |
|  | Abate et al., 2020, |  | Title and abstract |
|  | Asrat et al., 2020, |  | Title and abstract |
|  | Bekele et al., 2022, |  | Title and abstract |
|  | Adal et al., 2018, |  | Title and abstract |
|  | Oumer et al., 2019, |  | Title and abstract |
|  | Manyazewal et al., 2023 |  | Title and abstract |
|  | Alene et al., 2019, |  | Title and abstract |
|  | López et al., 2003, |  | Title and abstract |
|  | Ekubagewargies et al., 2019, |  | Title and abstract |
|  | Chibanda et al., 2020, |  | Title |
|  | Jemere and Kefale, 2021, |  | Title and abstract |
|  | Gaym, 2006, |  | Title and abstract |
|  | Keflie and Ameni, 2014, |  | Title and abstract |
|  | Awoke et al., 2016 |  | Title and abstract |
|  | Belayneh, et al, 2015 | Included |  |
|  | Lelisho et al., 2023, |  | Title and abstract |
|  | Geleso, 2020,., |  | Title and abstract |
|  | Wotale et al., 2021, |  | Title and abstract |
|  | Salomon and Murray, 2001, |  | Title and abstract |
|  | Zeleke and Zemedu, 2023, |  | Title and abstract |
|  | Worku et al., 2022, |  | Title and abstract |
|  | Bane et al., 2003, |  | Title and abstract |
|  | Getahun et al., 2011 |  | Title and abstract |
|  | Wubneh and Belay, 2020, |  | Title and abstract |
|  | Tesfayohannes et al., 2022, |  | Title and abstract |
|  | Biset Ayalew, 2017, |  | Title and abstract |
|  | Berhane et al., 2009, |  | Title and abstract |
|  | Angamo et al 2018a, |  | Title and abstract |
|  | Angamo et al., 2018b, |  | Title and abstract |
|  | Datiko and Lindtjørn, 2010, , |  | Title and abstract |
|  | Lubart et al., 2007, |  | Title and abstract |
|  | Alebel et al., 2020, |  | Title and abstract |
|  | Ali, et al, 2016 | Included |  |
|  | Gemechu et al., 2009, |  | Title and abstract |
|  | Belachew et al., 2022, |  | Title and abstract |
|  | Negesse et al., 2021, |  | Title and abstract |
|  | Birlie et al., 2017, |  | Title and abstract |
|  | Beyen, et al 2016 | Included |  |
|  | Diriba et al., 2021a, |  | Title and abstract |
|  | Misganaw et al., 2017b, |  | Title and abstract |
|  | Misganaw et al., 2017a, |  | Title and abstract |
|  | Tola et al., 2021, |  | Title and abstract |
|  | Law and Floyd, 2020, |  | Title and abstract |
|  | Herrero et al., 2009, |  | Title and abstract |
|  | Hibstie et al., 2020 |  | Title and abstract |
|  | Abdulla et al., 2023, , |  | Title and abstract |
|  | Diaz et al., 2005, |  | Title and abstract |
|  | Smitson et al., 2014, |  | Title and abstract |
|  | Berr et al., 2021, |  | Title and abstract |
|  | Hussen et al., 2016, |  | Title and abstract |
|  | Bekele et al., 2013 |  | Title and abstract |
|  | Mariam et al., 2008, |  | Title and abstract |
|  | Andargachew et al., 2008, |  | Title and abstract |
|  | Negash et al., 2020, |  | Title and abstract |
|  | Asfawesen et al., 2011, |  | Title and abstract |
|  | Bendayan et al., 2011 |  | Title and abstract |
|  | Balcha et al., 2015, |  | Title and abstract |
|  | Wagnew et al., 2018 |  | Title and abstract |
|  | Gele et al., 2009, |  | Title and abstract |
|  | Melaku et al., 2013, |  | Title and abstract |
|  | Demissie et al., 2002, |  | Title and abstract |
|  | Arja et al., 2021 |  | Title and abstract |
|  | Abdu et al., 2020, |  | Title and abstract |
|  | Adissu et al., 2020, |  | Title and abstract |
|  | Berhe et al., 2012, |  | Title and abstract |
|  | Sisay et al., 2019, |  | Title and abstract |
|  | Hodes and Seyoum, 1989, |  | Title and abstract |
|  | Hailu et al., 2023) |  | Title and abstract |
|  | Misganaw et al., 2012, |  | Title and abstract |
|  | Mulusew Asemahagn et al., 2018, , |  | Title and abstract |
|  | Menberu, 2016, |  | Title and abstract |
|  | Gashaw et al., 2021, |  | Title and abstract |
|  | G/Mariam, et al, 2016 | Included |  |
|  | Girma et al., 2023, |  | Title and abstract |
|  | Aderaye et al., 1996, |  | Title and abstract |
|  | Reepalu, et al, 2017 | Included |  |
|  | Koricho et al., 2010, |  | Title and abstract |
|  | Mengesha et al., 2022a, |  | Title and abstract |
|  | Alemu et al., 2020a |  | Title and abstract |
|  | Comas et al., 2015, |  | Title and abstract |
|  | Ifa, 2018 | Included |  |
|  | Tesfamariam et al., 2016, |  | Title and abstract |
|  | Mekonnen et al., 2023, |  | Title and abstract |
|  | Tiyou et al., 2010 |  | Title and abstract |
|  | Haile et al., 2014, |  | Title and abstract |
|  | Solomon et al., 2019, |  | Title and abstract |
|  | Asefa et al., 2019, |  | Title and abstract |
|  | Oumer et al., 2021, |  | Title and abstract |
|  | Gedfew, 2021, |  | Title and abstract |
|  | Haileamlak et al., 2017 |  | Title and abstract |
|  | Gebremichael et al., 2021, |  | Title and abstract |
|  | Setegn et al., 2015, , |  | Title and abstract |
|  | Birhanu et al., 2021, |  | Title and abstract |
|  | Tekelehaimanot et al., 2021, |  | Title and abstract |
|  | Misgina et al., 2019, |  | Title and abstract |
|  | Koye et al., 2012, |  | Title and abstract |
|  | Kassa et al., 2020b, |  | Title and abstract |
|  | Tola, et al,2019 | Included |  |
|  | Kassa et al., 2020a, |  | Title and abstract |
|  | Gemechu et al., 2022 |  | Title and abstract |
|  | Sileshi et al., 2013, |  | Title and abstract |
|  | Deribe et al., 2015a, |  | Title and abstract |
|  | Deribe et al., 2015b, |  | Title and abstract |
|  | Woldeyohannes et al., 2021 |  | Title and abstract |
|  | Gesesew et al., 2016, |  | Title and abstract |
|  | Workie et al., 2021, |  | Title and abstract |
|  | Gebremichael et al., 2018b |  | Title and abstract |
|  | Gebremichael et al., 2018a, |  | Title and abstract |
|  | Woldie et al., 2021, |  | Title and abstract |
|  | Mengesha et al., 2014, |  | Title and abstract |
|  | Siraj et al., 2022 |  | Title and abstract |
|  | Enderis et al., 2019, , |  | Title and abstract |
|  | Tekalegn et al., 2020, |  | Title and abstract |
|  | Hussen et al., 2019, |  | Title and abstract |
|  | Haile et al., 2016, |  | Title and abstract |
|  | Harrison et al., 2021, |  | Title and abstract |
|  | Gazeley et al., 2023, |  | Title and abstract |
|  | Debelu et al., 2021 |  | Title and abstract |
|  | Mohammedhussein et al., 2020, |  | Title and abstract |
|  | Mitku et al., 2016, |  | Title and abstract |
|  | Ayele et al., 2018, |  | Title and abstract |
|  | Assefa et al., 2015, |  | Title |
|  | Tesfaw et al., 2016, |  | Title and abstract |
|  | Duko et al., 2015, |  | Title and abstract |
|  | Woldegeorgis et al., 2023, |  | Title and abstract |
|  | Kiros et al., 2021 |  | Title and abstract |
|  | Bayisa et al., 2020, |  | Title and abstract |
|  | Schönfeld et al., 2018, |  | Title and abstract |
|  | Abseno et al., 2002, |  | Title and abstract |
|  | Bosho et al., 2018, |  | Title and abstract |
|  | Getinet et al., 2015, |  | Title and abstract |
|  | Mera et al., 2023, |  | Title and abstract |
|  | Menna et al., 2014, |  | Title and abstract |
|  | Aynalem et al., 2020, |  | Title and abstract |
|  | Woldeamanuel and Wondimu, 2018, |  | Title and abstract |
|  | Geliso, et al,2020 | Included |  |
|  | Woldeyes et al., 2022, |  | Title and abstract |
|  | Jemal et al., 2021 |  | Title and abstract |
|  | Abdisa et al., 2021, |  | Title and abstract |
|  | Sachithananthan et al., 2013, |  | Title and abstract |
|  | Reta et al., 2021 |  | Title and abstract |
|  | Umer et al., 2023, |  | Title and abstract |
|  | Alemayehu et al., 2017, |  | Title and abstract |
|  | Badacho and Mahomed, 2023, |  | Title and abstract |
|  | Bayleyegn et al., 2021b, |  | Title and abstract |
|  | Mulu et al., 2016, |  | Title and abstract |
|  | Diriba and Churiso, 2022, |  | Title and abstract |
|  | Mama et al., 2018, |  | Title and abstract |
|  | Wondimeneh et al., 2012, , |  | Title and abstract |
|  | Gebre and Mimano, 2010, |  | Title and abstract |
|  | Wolday and Messele, 2003 |  | Title and abstract |
|  | Meaza et al., 2023, |  | Title and abstract |
|  | Ababu et al., 2022, |  | Title and abstract |
|  | Gebretsadik et al., 2020, |  | Title and abstract |
|  | Muluye et al., 2013, |  | Title and abstract |
|  | Mitiku et al., 2023, |  | Title and abstract |
|  | Toru et al., 2022, |  | Title and abstract |
|  | Liyew Ayalew et al., 2020 |  | Title and abstract |
|  | Mirkuzie et al., 2021, |  | Title and abstract |
|  | Getaye et al., 2021, |  | Title and abstract |
|  | Tibebu and Hebo, 2019, |  | Title and abstract |
|  | Damtew et al., 2013, |  | Title and abstract |
|  | Yadeta et al., 2013, |  | Title and abstract |
|  | Asemahagn et al., 2020 |  | Title and abstract |
|  | Birhanu et al., 2021b, |  | Title and abstract |
|  | Anlay et al., 2016, |  | Title and abstract |
|  | Bitew et al., 2020, |  | Title |
|  | Mekuria et al., 2016, |  | Title and abstract |
|  | Woldegiyorgis and Scherrer, 2012, |  | Title and abstract |
|  | Feleke et al., 2013. |  | Title and abstract |
|  | Isaacson and Melaku, 2016, |  | Title and abstract |
|  | Abejew et al., 2014, |  | Title and abstract |
|  | Saravanan et al., 2018, |  | Title and abstract |
|  | Seyoum et al., 2017, |  | Title and abstract |
|  | Ketema et al., 2020, |  | Title and abstract |
|  | Bofe, 2022, |  | Title and abstract |
|  | Shimeles et al.,2019 |  | Title and abstract |
|  | Amante and Ahemed, 2015, |  | Title and abstract |
|  | Tsehay, 2019, |  | Title and abstract |
|  | Araya et al., 2018 |  | Title and abstract |
|  | Kebede et al., 2020b |  | Title and abstract |
|  | Gesesew et al., 2016a, |  | Title and abstract |
|  | Gidey et al., 2023, |  | Title and abstract |
|  | Schulman et al., 2019, |  | Title and abstract |
|  | Khogali et al., 2014, |  | Title and abstract |
|  | Binegdie et al., 2015, |  | Title and abstract |
|  | Weldemhret et al., 2016 |  | Title and abstract |
|  | Kasew et al., 2022, |  | Title and abstract |
|  | Wondimu, et al, 2020 | Included |  |
|  | Kampe et al., 2023 |  | Title and abstract |
|  | Abate and Wolde, 2016 |  | Title and abstract |
|  | Endris et al., 2015, |  | Title and abstract |
|  | Tigabu et al., 2019, |  | Title and abstract |
|  | Kebede, 2022, |  | Title and abstract |
|  | Gezae et al., 2023, |  | Title and abstract |
|  | Habte et al., 2019, |  | Title and abstract |
|  | Ramos et al., 2020, |  | Title and abstract |
|  | Amare et al., 2013, |  | Title and abstract |
|  | Yohanes et al., 2012, |  | Title and abstract |
|  | Gemechu and Debusho, 2023, |  | Title and abstract |
|  | Woya et al., 2019, |  | Title and abstract |
|  | Fidèle and Amanuel, 2016 |  | Title and abstract |
|  | Getaneh et al., 2023b, |  | Title and abstract |
|  | Haile et al., 2020, |  | Title and abstract |
|  | Hussien et al., 2022, |  | Title and abstract |
|  | Belay et al., 2014, |  | Title and abstract |
|  | Gizachew et al., 2021,) |  | Title and abstract |
|  | Sime, et al. 2022 | Included |  |
|  | Bitew et al., 2016, |  | Title and abstract |
|  | Gebreegziabiher et al., 2017, |  | Title and abstract |
|  | Salih et al., 2023, |  | Title and abstract |
|  | Teshale and Awoke, 2022, |  | Title and abstract |
|  | Kaso et al., 2022a, |  | Title and abstract |
|  | Gebreyes, 2023, |  | Title and abstract |
|  | Limenih and Workie, 2019, |  | Title and abstract |
|  | Damtew et al., 2015, |  | Title and abstract |
|  | Seyoum, et al, 2022 | Included |  |
|  | Nigussie et al., 2020, et al., 2021, |  | Title and abstract |
|  | Getaneh et al., 2022b, |  | Title and abstract |
|  | Abuto et al., 2021, |  | Title and abstract |
|  | Biyazin et al., 2022, |  | Title and abstract |
|  | Erdaw and Gobena, 2016, |  | Title and abstract |
|  | Tachbele and Ameni, 2016, |  | Title and abstract |
|  | Tolossa et al., 2021, |  | Title and abstract |
|  | Teka et al., 2021, Zinabu |  | Title and abstract |
|  | Fekadu, et al, 2022 | Included |  |
|  | Bade and Mega, 2020 |  | Title and abstract |
|  | Alemu, et al, 2021 | Included |  |
|  | (Muluneh et al., 2021, |  | Title and abstract |
|  | Getahun et al., 2023a, |  | Title and abstract |
|  | Deres et al., 2021, , |  | Title and abstract |
|  | Alemu et al., 2022a |  | Title and abstract |
|  | Liddle et al., 2013, |  | Title and abstract |
|  | Mekonnen et al., 2015 |  | Title and abstract |
|  | Teshome, et al, 2017 | Included |  |
|  | Woldeyohannes et al., 2011, |  | Title and abstract |
|  | Talargia and Getacher, 2021 |  | Title and abstract |
|  | Hussen Kabthymer et al., 2020, |  | Title and abstract |
|  | Birlie et al., 2015, |  | Title and abstract |
|  | Jabir et al., 2022, |  | Title and abstract |
|  | Palme, et al,2002 | Included |  |
|  | Mekonnen et al., 2023a, |  | Title and abstract |
|  | Asgedom et al., 2018 |  | Title and abstract |
|  | Tadege, 2018 |  | Title and abstract |
|  | Belay et al., 2022, |  | Title and abstract |
|  | Kebede et al., 2021a, |  | Title and abstract |
|  | Manaye et al., 2020b, |  | Title and abstract |
|  | Yimer et al., 2014, |  | Title and abstract |
|  | Zenebe et al., 2021, |  | Title and abstract |
|  | Amera et al., 2021, |  | Title and abstract |
|  | Alemu et al., 2022b, |  | Title and abstract |
|  | Ketema et al., 2019, |  | Title and abstract |
|  | Tsegaye et al., 2022 |  | Title and abstract |
|  | Zegeye et al., 2023, |  | Title and abstract |
|  | Wondim et al., 2020, |  | Title and abstract |
|  | Sinshaw, et al 2017 | Included |  |
|  | Ali and Yirtaw, 2019 |  | Title and abstract |
|  | Erjino et al., 2023, |  | Title and abstract |
|  | Mekuria et al., 2023, |  | Title and abstract |
|  | Balcha, et al, 2015 | Included |  |
|  | Marie et al., 2022, |  | Title and abstract |
|  | Weldemariam et al., 2022, |  | Title and abstract |
|  | Masresha et al., 2022, |  | Title and abstract |
|  | Asefa and Teshome, 2014, |  | Title and abstract |
|  | Awoke et al., 2019, |  | Title and abstract |
|  | Assefa et al., 2010, |  | Title and abstract |
|  | Seidu et al., 2023, |  | Title and abstract |
|  | Wagnew et al., 2019 |  | Title and abstract |
|  | Tefera et al., 2019, |  | Title and abstract |
|  | Adegeh, et al, 2021 | Included |  |
|  | Wedajo et al., 2022, |  | Title and abstract |
|  | Mezemir et al., 2022, |  | Title and abstract |
|  | Muñoz-Sellart et al., 2009, |  | Title and abstract |
|  | Belayneh et al., 2015a, |  | Title and abstract |
|  | Belayneh et al., 2015b, |  | Title and abstract |
|  | Omer et al., 2022, |  | Title and abstract |
|  | Weldegebreal et al., 2022, |  | Title and abstract |
|  | Weldegebreal et al., 2018a, |  | Title and abstract |
|  | Tola et al., 2019, |  | Title and abstract |
|  | H/Giorgis, et al.2018 | Included |  |
|  | Woldesemayat and Azeze, 2021, |  | Title and abstract |
|  | Endris et al., 2014 |  | Title and abstract |
|  | Tessema et al., 2009a, |  | Title and abstract |
|  | Tessema et al., 2009b, |  | Title and abstract |
|  | Getahun et al., 2013, |  | Title and abstract |
|  | Aye et al., 2023, |  | Title and abstract |
|  | Agazhu et al., 2023, , |  | Title and abstract |
|  | Adem et al., 2020, |  | Title and abstract |
|  | Fikrie et al., 2019a, |  | Title and abstract |
|  | Fikrie et al., 2019b |  | Title and abstract |
|  | Mamo et al., 2020, |  | Title and abstract |
|  | Ayele et al., 2015 |  | Title and abstract |
|  | Alene et al., 2017a, |  | Title and abstract |
|  | Alene et al., 2017b, |  | Title and abstract |
|  | Tilahun and Gebre-Selassie, 2016, |  | Title and abstract |
|  | Ereso et al., 2021 |  | Title and abstract |
|  | Wakjira et al., 2022, |  | Title and abstract |
|  | Worku et al., 2018, |  | Title and abstract |
|  | Kassa, et al, 2012 | Included |  |
|  | Gebreyohannes et al., 2018, |  | Title and abstract |
|  | Ashenafi et al., 2017, |  | Title and abstract |
|  | Dheresa et al., 2021, |  | Title and abstract |
|  | Alemu and Gutema, 2019, |  | Title and abstract |
|  | Adem et al., 2011, |  | Title and abstract |
|  | Girum et al., 2018c, |  | Title and abstract |
|  | Menna et al., 2015, |  | Title and abstract |
|  | Lelisho, et al,2023 | Included |  |
|  | Tessema et al., 2017 |  | Title and abstract |
|  | Hamusse et al., 2014a, |  | Title and abstract |
|  | Dememew et al., 2016, |  | Title and abstract |
|  | Dangisso et al., 2014, |  | Title and abstract |
|  | Hasen Badeso et al., 2022, |  | Title and abstract |
|  | Zenbaba et al., 2021, |  | Title and abstract |
|  | Deribew et al., 2016a, |  | Title and abstract |
|  | Deribew et al., 2016b, |  | Title and abstract |
|  | Legesse et al., 2021 |  | Title and abstract |
|  | Kebede et al., 2017, |  | Title and abstract |
|  | Alemu et al., 2021, |  | Title and abstract |
|  | Geremew et al., 2023a, |  | Title and abstract |
|  | Deribew et al., 2018, |  | Title and abstract |
|  | Wartski, 1993, |  | Title and abstract |
|  | Amsalu et al., 2007, |  | Title and abstract |
|  | van Griensven et al., 2018, |  | Title and abstract |
|  | Walles et al., 2022, |  | Title and abstract |
|  | Mohammed et al., 2021, |  | Title and abstract |
|  | Datiko and Lindtjørn, 2009, |  | Title, abstract , and study area |
|  | Berhan et al., 2023, |  | Title and abstract |
|  | Teferi et al., 2021 |  | Title and abstract |
|  | Ma et al., 2019 |  | Title and abstract |
|  | Getie and Alemnew, 2020,. |  | Title and abstract |
|  | Eshetie et al., 2018, |  | Title and abstract |
|  | Shaweno and Worku, 2012, |  | Title and abstract |
|  | G. Mariam A 2005, |  | Title and abstract |
|  | Yosha et al., 2021, |  | Title and abstract |
|  | Dheresa et al., 2022, |  | Title and abstract |
|  | Gebru et al., 2020, |  | Title and abstract |
|  | Muse et al., 2021, |  | Title and abstract |
|  | Shifera et al., 2022, |  | Title and abstract |
|  | Dessalegn et al., 2021, |  | Title and abstract |
|  | Seid and Ayele, 2020, |  | Title and abstract |
|  | Tiruneh et al., 2022, |  | Title and abstract |
|  | Girum et al., 2020a, |  | Title and abstract |
|  | Arega et al., 2020, |  | Title and abstract |
|  | Waju et al., 2021, |  | Title and abstract |
|  | Ambaw et al., 2018, |  | Title and abstract |
|  | Lulu and Berhane, 2005, |  | Title and abstract |
|  | Bisrat et al., 2022, |  | Title and abstract |
|  | Misganaw et al., 2012a, |  | Title and abstract |
|  | Kebede et al., 2021b, |  | Title and abstract |
|  | Fuge et al., 2021, |  | Title and abstract |
|  | Aseffa, 1993, |  | Title and abstract |
|  | Tafere et al., 2023, |  | Title and abstract |
|  | Hailu et al., 2018, |  | Title and abstract |
|  | Lyons et al., 2003, |  | Title and abstract |
|  | Getachew-Mebrahtu et al., 2017, |  | Title and abstract |
|  | Welay et al., 2016, |  | Title and abstract |
|  | Workineh et al., 2017, |  | Title and abstract |
|  | Gebrehiwet et al., 2023, |  | Title and abstract |
|  | Haftu et al., 2017, |  | Title and abstract |
|  | Mafirakureva et al., 2022, |  | Title and abstract |
|  | Ayalew et al., 2023 |  | Title and abstract |
|  | Wendwessen et al., 2020, |  | Title and abstract |
|  | Fenta and Nigussie, 2021a, 0, |  | Title and abstract |
|  | Bedaso et al., 2018, |  | Title and abstract |
|  | Dagnew et al., 2020 |  | Title and abstract |
|  | Paulose et al., 2022, |  | Title and abstract |
|  | Debere et al., 2022, |  | Title and abstract |
|  | Abdi et al., 2020, |  | Title and abstract |
|  | Lambebo et al., 2021, |  | Title and abstract |
|  | Ersino et al., 2018, |  | Title and abstract |
|  | Degu et al., 2023, |  | Title and abstract |
|  | Chekol et al., 2023, |  | Title and abstract |
|  | Atnafu et al., 2020, |  | Title and abstract |
|  | Chilot et al., 2023, |  | Title and abstract |
|  | Terefe et al., 2023, |  | Title and abstract |
|  | Hajare et al., 2022 |  | Title and abstract |
|  | Forouzanfar et al., 2017, |  | Title and abstract |
|  | Fitzmaurice et al., 2018, |  | Title, abstract ,and study area |
|  | Kumar et al.,2022 |  | Title and abstract |
|  | Sekaran et al., 2023, |  | Title and abstract |
|  | Begashaw et al., 2016, |  | Title and abstract |
|  | Kefyalew et al., 2016, |  | Title and abstract |
|  | Gizalew Snr et al., 2021, |  | Title and abstract |
|  | Alemu et al., 2020d, |  | Title and abstract |
|  | Gelaw et al., 2023, |  | Title and abstract |
|  | Getaneh et al., 2020, |  | Title and abstract |
|  | Endalamaw et al., 2019, |  | Title and abstract |
|  | Muluneh et al., 2022, |  | Title and abstract |
|  | Geremew et al., 2021, |  | Title and abstract |
|  | Regassa and Stoecker, 2012, |  | Title and abstract |
|  | Ruducha et al., 2017, , |  | Title and abstract |
|  | Gopalan et al., 2022, |  | Title and abstract |
|  | Endriyas et al., 2023a, |  | Title and abstract |
|  | Mason et al., 2010, |  | Title and abstract |
|  | Argaw et al., 2022, |  | Title and abstract |
|  | Sully et al., 2023, |  | Title, abstract, and Setting. |
|  | Konings et al., 2012 |  | Title and abstract |
|  | Manyazewal et al., 2018, |  | Title and abstract |
|  | Fenta and Nigussie, 2021b, |  | Title and abstract |
|  | Assefa et al., 2020, |  | Title and abstract |
|  | Bogale et al., 2009, |  | Title and abstract |
|  | Medhanyie et al., 2019 |  | Title and abstract |
|  | Abate et al., 2022, |  | Title and abstract |
|  | Endriyas et al., 2021, |  | Title and abstract |
|  | Endriyas et al., 2023b, |  | Title and abstract |
|  | Gilano et al., 2022, |  | Title and abstract |
|  | Woyessa et al., 2022, |  | Title and abstract |
|  | Goshu Muluneh et al., 2022, |  | Title and abstract |
|  | Issa, 2021, Buda et al., 2017) |  | Title and abstract |
|  | Shiferaw et al., 2012, |  | Title and abstract |
|  | Denur et al., 2019, |  | Title and abstract |
|  | Habte and Demissie, 2015, |  | Title and abstract |
|  | Hailemichael et al., 2015, |  | Title |
|  | Tekalign et al., 2021, |  | Title and abstract |
|  | Alene et al., 2020b, |  | Title and abstract |
|  | Negash et al., 2005, |  | Title and abstract |
|  | Shargie et al., 2008, |  | Title and abstract |
|  | Haidar and Demissie, 1999, |  | Title and abstract |
|  | Asmare et al., 2016, |  | Title and abstract |
|  | Molla and Delil, 2015, |  | Title and abstract |
|  | Atalell et al., 2022 |  | Title and abstract |
|  | Nedamo et al., 2023, |  | Title and abstract |
|  | Kumari et al., 2022, |  | Title and abstract |
|  | Erjino et al., 2022, |  | Title |
|  | Berhan, 2008, |  | Title and abstract |
|  | Reta et al., 2020, |  | Title and abstract |
|  | Girma et al., 2023a, |  | Title and abstract |
|  | Molina-Moya et al., 2018, |  | Title and abstract |
|  | Tamrat et al., 2020, |  | Title and abstract |
|  | Fetene et al., 2022, |  | Title and abstract |
|  | Kassa, 2018, |  | Title and abstract |
|  | Ludwick et al., 2022, |  | Title, abstract , and study area |
|  | Gebre et al., 2022, |  | Title and abstract |
|  | Gelan et al., 2023, |  | Title and abstract |
|  | Muhamed et al., 2022 |  | Title and abstract |
|  | Tesfaye et al., 2019, |  | Title and abstract |
|  | Tesfaye et al., 2020c |  | Title and abstract |
|  | Assefa et al., 2016b, |  | Title and abstract |
|  | Lrago et al., 2018, |  | Title |
|  | Birhanu et al., 2023, |  | Title and abstract |
|  | Endriyas et al., 2018, |  | Title and abstract |
|  | Aynalem et al., 2022, |  | Title and abstract |
|  | Bedaso et al., 2020b, |  | Title and abstract |
|  | Bekele et al., 2022b, |  | Title and abstract |
|  | Gemechu et al., 2022a, |  | Title and abstract |
|  | Menna, T. et al.,2015 |  | Title and abstract |
|  | Bedaso and Ayalew, 2019, |  | Title and abstract |
|  | Roro et al., 2022, |  | Title and study area |
|  | Girma and Aemiro, 2022, |  | Title and abstract |
|  | Tadesse et al., 2019, , |  | Title and abstract |
|  | Alemu et al., 2020d, |  | Title and abstract |
|  | Hagos and Tadesse, 2020, |  | Title |
|  | Negussie et al., 2018, |  | Title and abstract |
|  | Sonko and Worku, 2015, |  | Title and abstract |
|  | Haftom et al., 2021, |  | Title and abstract |
|  | Waja et al., 2016, |  | Title and abstract |
|  | Adera et al., 2016, |  | Title and abstract |
|  | Hajare et al., 2022b, |  | Title |
|  | Yosef et al., 2023, |  | Title and abstract |
|  | Abate et al., 2023, |  | Title and abstract |
|  | Paulose et al., 2022b, |  | Title and abstract |
|  | Debo and Kassa, 2016, |  | Title and abstract |
|  | Otayto et al., 2022 |  | Title, abstract, and setting |
|  | Hajare et al., 2021, |  | Title, abstract , setting |
|  | Kendie et al., 2021, |  | Title and abstract |
|  | Awol et al., 2021, , |  | Title and abstract |
|  | Bisetegn et al., 2021, |  | Title and abstract |
|  | Hailegebriel et al., 2020, |  | Title and abstract |
|  | Chelkeba et al., 2022, |  | Title and abstract |
|  | Shewarega et al., 2023, |  | Title and abstract |
|  | Kebede et al., 2021a, |  | Title and abstract |
|  | Mengesha Kassie et al., 2020, |  | Title and abstract |
|  | Muhamed et al., 2023, |  | Title and abstract |
|  | Abamecha et al., 2019, |  | Title and abstract |
|  | Olapeju et al., 2022, |  | Title and abstract |
|  | Hrusa et al., 2020 |  | Title, abstract and study area |
|  | Bedaso et al., 2020a, |  | Title and abstract |
|  | Caprioli et al., 2020 |  | Title and abstract |
|  | Gelgie et al., 2022, |  | Title and abstract |
|  | Endriyas et al., 2023c, |  | Title and abstract |
|  | Dejene et al., 2021, |  | Title and abstract |
|  | De Smet and Boroş, 2021, |  | Title, abstract, and study area |
|  | Takele et al., 2019, |  | Title and abstract |
|  | Admassu et al., 2023, |  | Title and abstract |
|  | Bedru et al., 2022, |  | Title and abstract |
|  | Abebe and Gatisso, 2023, |  | Title and abstract |
|  | Aregu et al., 2021, |  | Title and abstract |
|  | Lerango et al., 2023, |  | Title and abstract |
|  | Mekonnen et al., 2019, |  | Title and abstract |
|  | Belay et al., 2022b, |  | Title and abstract |
|  | Belay et al., 2022c, |  | Title and abstract |
|  | Chernet et al., 2017, |  | Title and abstract |
|  | Habte et al., 2019b) |  | Title and abstract |
|  | Fekadu and Regassa, 2014 |  | Title and abstract |
|  | Reta et al., 2020a, |  | Title and abstract |
|  | Demsash et al., 2023, |  | Title |
|  | Terefe et al., 2022, |  | Title and abstract |
|  | Tesfa et al., 2022, , |  | Title and abstract |
|  | Aragaw et al., 2023c |  | Title and abstract |
|  | Kassie et al., 2022 |  | Title and abstract |
|  | Tessema and Zeleke, 2020, |  | Title and abstract |
|  | Tesfa et al., 2023, |  | Title and abstract |
|  | Aragaw et al., 2023a, |  | Title |
|  | Melaku et al., 2020a, |  | Title and abstract |
|  | Tusa et al., 2021, |  | Title and abstract |
|  | Asmamaw et al., 2023, |  | Title and abstract |
|  | Tessema and Akalu, 2020, |  | Title and abstract |
|  | Warkaw et al., 2022, |  | Title |
|  | Sisay et al., 2019b, , |  | Title and abstract |
|  | Hailegebreal et al., 2022 |  | Title and abstract |
|  | Teshale et al., 2021a, |  | Title and abstract |
|  | Aragaw et al., 2023b, |  | Title and abstract |
|  | Merid et al., 2023, |  | Title and abstract |
|  | Beyene et al., 2018, |  | Title and abstract |
|  | Tessema and Tiruneh, |  | Title and abstract |
|  | 2020, Belay et al., 2021b, |  | Title and abstract |
|  | Delibo et al., 2020, |  | Title and abstract |
|  | Batisso et al., 2012, |  | Title |
|  | Ravisankar et al., 2018, |  | Title and abstract |
|  | Bayou et al., 2022, |  | Title |
|  | Chaka et al., 2018, B |  | Title and abstract |
|  | eketie et al., 2021, |  | Title and abstract |
|  | Zenebe et al., 2021b, |  | Title |
|  | Asefa et al., 2020, |  | Title and abstract |
|  | Ketema et al., 2011, |  | Title |
|  | Lemma Tirore et al., 2022, , |  | Title and abstract |
|  | Asfaw et al., 2020, |  | Title and abstract |
|  | Sekaran et al., 2023b, |  | Title and abstract |
|  | Broström et al., 2020, |  | Study area |
|  | Kebede et al., 2023a, |  | Title and abstract |
|  | Mohammed et al., 2017, |  | Title |
|  | Ergete et al., 2018, |  | Title and abstract |
|  | Kassaw et al., 2021, |  | Title and abstract |
|  | Amare, 2014, |  | Title and abstract |
|  | Smith et al., 2022, |  | Title and abstract |
|  | Endriyas et al., 2019,) |  | Title and abstract |
|  | Arora et al., 2020, |  | Title and abstract |
|  | Aragaw et al., 2023d, |  | Title and abstract |
|  | Wolde et al., 2020, |  | Title and abstract |
|  | Hailegebreal et al., 2023 |  | Title and abstract |
|  | Kloos et al., 2007, |  | Study area |

**After screening, the data were extracted by Wubet Tazeb Wondie and Belay Tafa Regassa**

**Date of data extraction November 26/2023 to December 26/2023.**

**Time from inception to November 25/2023.**
