# Supplementary material for: Mortality and predictors among HIV-TB co-infected patients in Ethiopia: A systematic review and meta-analysis
Source: PLoS One. 2025 Jan 6;20(1):e0317048. doi: 10.1371/journal.pone.0317048 (PMC11703055; doi:10.1371/journal.pone.0317048)
Supplement: S4 Table — (DOCX) [file pone.0317048.s004.docx]

S3 Table A: Quality assessment for included studies in meta-analysis for Cohort study

| Author, Year | Q1 | | | | Q2 | | | | Q3 | | | | Q4 | | | | Q5 | | | | Q6 | | | | Q7 | | | | Q8 | | | | Q9 | | | | Q10 | | | | Q11 | | | | Overall quality |
| --- | --- | --- | --- | --- | --- | --- | --- | --- | --- | --- | --- | --- | --- | --- | --- | --- | --- | --- | --- | --- | --- | --- | --- | --- | --- | --- | --- | --- | --- | --- | --- | --- | --- | --- | --- | --- | --- | --- | --- | --- | --- | --- | --- | --- | --- |
|  | Y | N | U | NA | Y | N | U | NA | Y | N | U | NA | Y | N | U | NA | Y | N | U | NA | Y | N | U | NA | Y | N | U | NA | Y | N | U | NA | Y | N | U | NA | Y | N | U | NA | Y | N | U | NA |  |
| Dawit Z et al, 2021 |  |  |  | **√** |  |  |  | **√** | **√** |  |  |  | **√** |  |  |  | **√** |  |  |  | **√** |  |  |  | **√** |  |  |  | **√** |  |  |  | **√** |  |  |  | **√** |  |  |  | **√** |  |  |  | 9/11(81.81%) |
| Gemechu et al,2022 |  |  |  | **√** |  |  |  | **√** | **√** |  |  |  | **√** |  |  |  | **√** |  |  |  | **√** |  |  |  | **√** |  |  |  | **√** |  |  |  | **√** |  |  |  | **√** |  |  |  | **√** |  |  |  | 9/11(81.81%) |
| Atallel et al 2018 |  |  |  | **√** |  |  |  | **√** | **√** |  |  |  | **√** |  |  |  | **√** |  |  |  | **√** |  |  |  | **√** |  |  |  | **√** |  |  |  | **√** |  |  |  | **√** |  |  |  | **√** |  |  |  | 9/11(81.81%) |
| Chanie et al, 2021 |  |  |  | **√** |  |  |  | **√** | **√** |  |  |  | **√** |  |  |  | **√** |  |  |  | **√** |  |  |  | **√** |  |  |  | **√** |  |  |  | **√** |  |  |  | **√** |  |  |  | **√** |  |  |  | 9/11(81.81%) |
| Negussie et al,2021 |  |  |  | **√** |  |  |  | **√** | **√** |  |  |  | **√** |  |  |  | **√** |  |  |  | **√** |  |  |  | **√** |  |  |  | **√** |  |  |  | **√** |  |  |  | **√** |  |  |  | **√** |  |  |  | 9/11(81.81%) |
| Alula MT et al, 2017 | **√** |  |  |  | **√** |  |  |  | **√** |  |  |  | **√** |  |  |  | **√** |  |  |  | **√** |  |  |  | **√** |  |  |  | **√** |  |  |  | **√** |  |  |  | **√** |  |  |  | **√** |  |  |  | 11/11(100%) |
| Shaweno et al, 2012 |  |  |  | **√** |  |  |  | **√** | **√** |  |  |  | **√** |  |  |  | **√** |  |  |  | **√** |  |  |  | **√** |  |  |  | **√** |  |  |  | **√** |  |  |  | **√** |  |  |  | **√** |  |  |  | 9/11(81.81%) |
| Gebreyes, 2023 |  |  |  | **√** |  |  |  | **√** | **√** |  |  |  | **√** |  |  |  | **√** |  |  |  | **√** |  |  |  | **√** |  |  |  | **√** |  |  |  | **√** |  |  |  | **√** |  |  |  | **√** |  |  |  | 9/11(81.81%) |
| Habtamu et al, 2021 |  |  |  | **√** |  |  |  | **√** | **√** |  |  |  | **√** |  |  |  | **√** |  |  |  | **√** |  |  |  | **√** |  |  |  | **√** |  |  |  | **√** |  |  |  | **√** |  |  |  | **√** |  |  |  | 9/11(81.81%) |
| Abrha H et al, 2015 |  |  |  | **√** |  |  |  | **√** | **√** |  |  |  | **√** |  |  |  | **√** |  |  |  | **√** |  |  |  | **√** |  |  |  | **√** |  |  |  | **√** |  |  |  | **√** |  |  |  | **√** |  |  |  | 9/11(81.81%) |
| Birhan et al, 2021 |  |  |  | **√** |  |  |  | **√** | **√** |  |  |  | **√** |  |  |  | **√** |  |  |  | **√** |  |  |  | **√** |  |  |  | **√** |  |  |  | **√** |  |  |  | **√** |  |  |  | **√** |  |  |  | 9/11(81.81%) |
| Refera et al, 2013 |  |  |  | **√** |  |  |  | **√** | **√** |  |  |  | **√** |  |  |  | **√** |  |  |  | **√** |  |  |  | **√** |  |  |  | **√** |  |  |  | **√** |  |  |  | **√** |  |  |  | **√** |  |  |  | 9/11(81.81%) |
| Gezae, et al, 2019 |  |  |  | **√** |  |  |  | **√** | **√** |  |  |  | **√** |  |  |  | **√** |  |  |  | **√** |  |  |  | **√** |  |  |  | **√** |  |  |  | **√** |  |  |  | **√** |  |  |  | **√** |  |  |  | 9/11(81.81%) |
| Lelisho et al, 2022 |  |  |  | **√** |  |  |  | **√** | **√** |  |  |  | **√** |  |  |  | **√** |  |  |  | **√** |  |  |  | **√** |  |  |  | **√** |  |  |  | **√** |  |  |  | **√** |  |  |  | **√** |  |  |  | 9/11(81.81%) |
| Sileshi et al , 2013 |  |  |  | **√** |  |  |  | **√** | **√** |  |  |  | **√** |  |  |  | **√** |  |  |  | **√** |  |  |  | **√** |  |  |  | **√** |  |  |  | **√** |  |  |  | **√** |  |  |  | **√** |  |  |  | 9/11(81.81%) |
| Beyen, et al 2016 |  |  |  | **√** |  |  |  | **√** | **√** |  |  |  | **√** |  |  |  | **√** |  |  |  | **√** |  |  |  | **√** |  |  |  | **√** |  |  |  | **√** |  |  |  | **√** |  |  |  | **√** |  |  |  | 9/11(81.81%) |
| Reepalu et al, 2017 |  |  |  | **√** |  |  |  | **√** | **√** |  |  |  | **√** |  |  |  | **√** |  |  |  | **√** |  |  |  | **√** |  |  |  | **√** |  |  |  | **√** |  |  |  | **√** |  |  |  | **√** |  |  |  | 9/11(81.81%) |
| **Geliso, M.G. 2020** |  |  |  | **√** |  |  |  | **√** | **√** |  |  |  | **√** |  |  |  | **√** |  |  |  | **√** |  |  |  | **√** |  |  |  | **√** |  |  |  | **√** |  |  |  | **√** |  |  |  | **√** |  |  |  | 9/11(81.81%) |
| **Wondimu, et al, 2020** |  |  |  | **√** |  |  |  | **√** | **√** |  |  |  | **√** |  |  |  | **√** |  |  |  | **√** |  |  |  | **√** |  |  |  | **√** |  |  |  | **√** |  |  |  | **√** |  |  |  | **√** |  |  |  | 9/11(81.81%) |
| Sime T. et al. 2022 |  |  |  | **√** |  |  |  | **√** | **√** |  |  |  | **√** |  |  |  | **√** |  |  |  | **√** |  |  |  | **√** |  |  |  | **√** |  |  |  | **√** |  |  |  | **√** |  |  |  | **√** |  |  |  | 9/11(81.81%) |
| Seyoum et al, 2022 |  |  |  | **√** |  |  |  | **√** | **√** |  |  |  | **√** |  |  |  | **√** |  |  |  | **√** |  |  |  | **√** |  |  |  | **√** |  |  |  | **√** |  |  |  | **√** |  |  |  | **√** |  |  |  | 9/11(81.81%) |
| **Fekadu,et al, 2022** |  |  |  | **√** |  |  |  | **√** | **√** |  |  |  | **√** |  |  |  | **√** |  |  |  | **√** |  |  |  | **√** |  |  |  | **√** |  |  |  | **√** |  |  |  | **√** |  |  |  | **√** |  |  |  | 9/11(81.81%) |
| **Alemu, et al , 2021** |  |  |  | **√** |  |  |  | **√** | **√** |  |  |  | **√** |  |  |  | **√** |  |  |  | **√** |  |  |  | **√** |  |  |  | **√** |  |  |  | **√** |  |  |  | **√** |  |  |  | **√** |  |  |  | 9/11(81.81%) |
| Palme,I.M et al,2002 |  |  |  | **√** |  |  |  | **√** | **√** |  |  |  | **√** |  |  |  | **√** |  |  |  | **√** |  |  |  | **√** |  |  |  | **√** |  |  |  | **√** |  |  |  | **√** |  |  |  | **√** |  |  |  | 9/11(81.81%) |
| **Balcha, et al, 2015** |  |  |  | **√** |  |  |  | **√** | **√** |  |  |  | **√** |  |  |  | **√** |  |  |  | **√** |  |  |  | **√** |  |  |  | **√** |  |  |  | **√** |  |  |  | **√** |  |  |  | **√** |  |  |  | 9/11(81.81%) |
| **Adegeh et al, 2021** |  |  |  | **√** |  |  |  | **√** | **√** |  |  |  | **√** |  |  |  | **√** |  |  |  | **√** |  |  |  | **√** |  |  |  | **√** |  |  |  | **√** |  |  |  | **√** |  |  |  | **√** |  |  |  | 9/11(81.81%) |
| **H/Giorgis et al.2018** |  |  |  | **√** |  |  |  | **√** | **√** |  |  |  | **√** |  |  |  | **√** |  |  |  | **√** |  |  |  | **√** |  |  |  | **√** |  |  |  | **√** |  |  |  | **√** |  |  |  | **√** |  |  |  | 9/11(81.81%) |
| **Kassa A, et al, 2012** |  |  |  | **√** |  |  |  | **√** | **√** |  |  |  | **√** |  |  |  | **√** |  |  |  | **√** |  |  |  | **√** |  |  |  | **√** |  |  |  | **√** |  |  |  | **√** |  |  |  | **√** |  |  |  | 9/11(81.81%) |
| **Lelisho,et al,2023** |  |  |  | **√** |  |  |  | **√** | **√** |  |  |  | **√** |  |  |  | **√** |  |  |  | **√** |  |  |  | **√** |  |  |  | **√** |  |  |  | **√** |  |  |  | **√** |  |  |  | **√** |  |  |  | 9/11(81.81%) |

S3 Table B: Quality assessment for included studies in meta-analysis for cross-sectional design

| Author, Year | Q1 | | | Q2 | | | | Q3 | | | | Q4 | | | | Q5 | | | | Q6 | | | | Q7 | | | | Q8 | | | | Overall quality in % |
| --- | --- | --- | --- | --- | --- | --- | --- | --- | --- | --- | --- | --- | --- | --- | --- | --- | --- | --- | --- | --- | --- | --- | --- | --- | --- | --- | --- | --- | --- | --- | --- | --- |
|  | N | U | NA | Y | N | U | NA | Y | N | U | NA | Y | N | U | NA | Y | N | U | NA | Y | N | U | NA | Y | N | U | NA | Y | N | U | NA |  |
| W/Gebreal et al, 2018 | **√** |  |  | **√** |  |  |  | **√** |  |  |  | **√** |  |  |  | **√** |  |  |  | **√** |  |  |  | **√** |  |  |  | **√** |  |  |  | 8/8 (100) |
| Belayneh,et al, 2015 | **√** |  |  | **√** |  |  |  | **√** |  |  |  | **√** |  |  |  | **√** |  |  |  | **√** |  |  |  | **√** |  |  |  | **√** |  |  |  | 8/8 (100) |
| Ali, et al , 2016 | **√** |  |  | **√** |  |  |  | **√** |  |  |  |  |  | √ |  | **√** |  |  |  | **√** |  |  |  | **√** |  |  |  | **√** |  |  |  | 7/8 (87.5) |
| G/Mariam,et al, 2016 | **√** |  |  | **√** |  |  |  | **√** |  |  |  |  |  | **√** |  | **√** |  |  |  | **√** |  |  |  | **√** |  |  |  | **√** |  |  |  | 7/8 (87.5) |
| Ifa , 2018 | **√** |  |  | **√** |  |  |  | **√** |  |  |  | **√** |  |  |  | **√** |  |  |  | **√** |  |  |  | **√** |  |  |  | **√** |  |  |  | 8/8 (100) |
| Tola, et al,2019 | **√** |  |  | **√** |  |  |  | **√** |  |  |  | **√** |  |  |  | **√** |  |  |  | **√** |  |  |  | **√** |  |  |  | **√** |  |  |  | 8/8 (100) |
| **Teshome,et al, 2017** | **√** |  |  | **√** |  |  |  | **√** |  |  |  | **√** |  |  |  | **√** |  |  |  | **√** |  |  |  | **√** |  |  |  | **√** |  |  |  | 7/8 (87.5) |
| **Sinshaw et al 2017** | **√** |  |  | **√** |  |  |  | **√** |  |  |  |  |  | √ |  | **√** |  |  |  | **√** |  |  |  | **√** |  |  |  | **√** |  |  |  | 7/8 (87.5) |
| Adegeh et al, 2021 | **√** |  |  | **√** |  |  |  | **√** |  |  |  | **√** |  |  |  | **√** |  |  |  | **√** |  |  |  | **√** |  |  |  | **√** |  |  |  | 8/8 (100) |

*Y=yes, N=no, U=unclear, NA=not applicable, <60%=low, 60-80%=medium, >80%=high quality.
