# Supplementary figures and images for: Mortality and predictors among HIV-TB co-infected patients in Ethiopia: A systematic review and meta-analysis
Source: PLoS One. 2025 Jan 6;20(1):e0317048. doi: 10.1371/journal.pone.0317048 (PMC11703055; doi:10.1371/journal.pone.0317048)

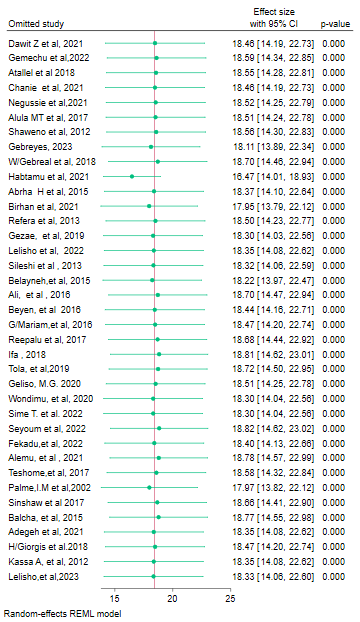

Supplement: S1 Fig — (TIF) [file pone.0317048.s006.tif]
