# Supplementary material for: Mortality and predictors among HIV-TB co-infected patients in Ethiopia: A systematic review and meta-analysis
Source: PLoS One. 2025 Jan 6;20(1):e0317048. doi: 10.1371/journal.pone.0317048 (PMC11703055; doi:10.1371/journal.pone.0317048)
Supplement: Attachment — Submitted filename: Response to Editor and Reviewers.docx [file pone.0317048.s007.docx]

**Authors' response to the Editor, and Reviewers' concern**

**Title: Mortality and predictors among HIV/TB co-infected patients in Ethiopia: A Systematic Review and Meta-analysis.**

**Manuscript ID: PONE-D-24-06919**

**Date: July, 10, 2024.**

**Subject: Revision of the manuscript.**

Thank you very much dear editor and reviewers for your valuable and constructive suggestions and comments. We found that the comments are very helpful and constructive to further improve the manuscript. This is our point-by-point response of authors to the editor and reviewers’ suggestions and concerns about the manuscript entitled “Mortality and predictors among HIV/TB co-infected patients in Ethiopia: A Systematic Review and Meta-analysis” which has a manuscript ID of “PONE-D-24-06919” given by the journal. It is known that the manuscript has been reviewed by reviewers and sent back to the authors for revision and resubmission. As authors of this manuscript, the comments and concerns raised by the reviewers and editor are highly important enabling us to improve the quality and plausibility of the manuscript. To do so, we have addressed all of the reviewer's concerns point by point. Therefore, we are very much pleased to resubmit the revised version of the manuscript for further revision process and facilitation of its publication on PLOS ONE.

We look forward to hear from you at your earliest convenience.

With best regards.

Corresponding Author

Wubet Tazeb Wondie	**E-mail:** wubettazeb27@gmail.com.

On behalf of Co-authors.

**Academic Editor’s comments and Authors’ response.**

**Concern 1:** When submitting your revision, we need you to address these additional requirements. Please ensure that your manuscript meets PLOS ONE's style requirements, including those for file naming.

**Authors Response:** Thank you dear editor for your concern. We have accepted and corrected in the revised manuscript.

**Concern 2:** We suggest you thoroughly copyedit your manuscript for language usage, spelling, and grammar. If you do not know anyone who can help you do this, you may wish to consider employing a professional scientific editing service.

**Authors Response:** Thank you dear editor for your concern. We have accepted and corrected in the revised manuscript as much as possible.

**Concern 3:** In the online submission form, you indicated that [All data analyzed and used during this study are available upon reasonable request].

All PLOS journals now require all data underlying the findings described in their manuscript to be freely available to other researchers, either 1. In a public repository, 2. Within the manuscript itself, or 3. Uploaded as supplementary information.

**Authors response:** Thank you for your suggestion. We have accepted and the data is freely available as supplementary information.

**Concern 4:** Please include captions for your Supporting Information files at the end of your manuscript, and update any in-text citations to match accordingly.

**Authors response:** Thank you dear editor for your suggestions. We have accepted and included cations of supporting information at the end of the manuscript.

**Point-by-point Response Letter**

Dear editorial office of PLOS ONE we have presented our point-by-point response in a way that the reviewers' concern is depicted first, and the authors' response has been given immediately next to it.

**Reviewer 1 concerns and Authors’ response.**

**Concern 1:** Abstract Cochrane’s I2 is a secondary confirmation, it does not provide a p-value. Use tau-squared.

Authors response: Thank you dear reviewer for your golden suggestion. We have accepted and corrected using STATA 18 in the revised manuscript based on your recommendation.

**Concern 2:** I2 should be stated as Cochrane’s I^2

**Authors response:** Thank you for your valuable suggestion. We have accepted and corrected through out the modified manuscript.

**Concern 3:** Pooled proportion is 18.35 (95% CI: 14.89-21.81) calculated as follows:

18.35+1.96σ x (s/√n) = 18.35+1.96.(10.73/√37) =21.81 upper limit

18.35-1.96σ x (s/√n) = 18.35-1.96.(10.73/√37) =14.89 lower limit

s = sample standard deviation; n = 37.

α = 0.025 x 37 = 0.925 ≈ 1.

This means that your funnel plot should not have more than 1 observation outside the left pseudo 95% limit and not more than 1 outside the right pseudo limit. You have 7 outside the left limit and 16 outside the right limit. This shows your distribution is highly non-normal, and that the parameters of a normal distribution do not apply. The funnel plot does not indicate that an Egger’s trim and fill has been done because the number of papers remains at 37, and the left half of the funnel is relatively empty. The funnel plot is thus unacceptable in all details.

**Authors response:** Thank you dear reviewer for your valuable and golden concern. As you have said in the random effects model using the Dersimonian Liard method, there are 7 studies out of the left limit and 16 studies out of the right limit, and the left side funnel plot is relatively empty, this suggests a high degree of heterogeneity. So, to assess the publication bias we have used the Eggers test for small study effect, and non-parametric trim and fill analysis. Accordingly, in the non-parametric trim and fill analysis 3 studies have been imputed and a total of 40 studies were included in the observed and imputed studies in the random effects Restricted Maximum Likelihood model. Due to the non-normal distribution of the data we have used non-parametric trim and fill analysis rather than the standard trim and fill analysis. In addition, we used a Galbraith plot to identify the presence of heterogeneity.

**Concern 3:** You have used STATA 14.2 for your meta-analysis, which is an add-in using the meta command. This uses the DerSimonian and Laird approach (DL) for the pooled effects and the diamond. You have overall DL at p<0.000 and I^2 =97.1%. This does not show the detailed tests for heterogeneity such as Tau-squared, H-squared and Q (see Borenstein below). STATA 16 onwards incorporates meta-analysis in its suite. I would recommend you use the latest version (STATA 18). Read also the STATA manual on meta-analysis. Note that funnel plots are rarely successful (Borenstein et.al. (2009)) mainly because of incomplete spread of publications.

Consult the paper: Michael Borenstein (8 November, 2023) https://doi.org/10.1002/jrsm.1678 Avoiding common mistakes in meta-analysis: Understanding the distinct roles of Q, I-squared, tau-squared, and the prediction interval in reporting heterogeneity (Wiley).

**Authors response:** Thank you dear reviewer for your golden suggestions. We have accepted and used the latest version STATA18 based on your recommendation, and we tried to show Tau-squared, H-squared, and Q heterogeneity tests

**Concern 4:** Figure 2 Forest plot of pooled mortality. Although the random effects model applies, and publications therefore have similar weights, the effects confirm the funnel plot’s conclusion of a very non-normal distribution. The top 5 effect means are: Habtamu 80.48; Palme et.al. 37.93; Birhan et.al. 35.39; Gebreyes 29.48; Belayneh et.al. 25.7. The bottom 5 effect means are: Ifa 4.40; Seyoum et.al. 4.45; Alemu et.al. 5.63; Balcha et.al. 6.15; Tola et.al. 7.70. Using the upper limit equation above 18.35+1.96*(10.73/√37) =21.81 upper limit and 18.35-1.96*(10.73/√37) =14.89 lower limit. In the top and bottom studies, Habtamu and Ifa respectively, we have:

The Habtamu mean has a z=35.2 above the mean, that is: 18.35+35.2*(10.73306/37^0.5) ≈ 80.48

The Ifa mean has a z=7.9 below the mean, that is 18.35-7.9*(10.73306/37^0.5) ≈ 4.40.

Applying these to the other 8 studies highlights the problems of the outliers in the funnel plot.

The conclusion on this forest plot is that the 37 studies constitute a highly non-normal distribution and that the 18.35% prevalence is invalid as a measure for Ethiopia as a whole.

**Authors response:** Thank you dear reviewer for your valuable concern. As you have said there are outliers Habtamu et, al, Ifa … The main reason for this outlier and variation is in the Tigray region there is poor adherence and awareness of ART which increases the mortality of HIV-infected patients particularly in co-infected patients that means the Habtamu’s study is higher than the other. On the other hand, in SNNPR, the community has a good awareness about ART and the importance of good adherence, which reduces the mortality rate, that means the Ifa et al study (a study conducted in SNNP), is lower than the other.

By considering the variation of those studies, we have used a random effect restricted maximum likelihood model. To identify the potential source of heterogeneity, a sensitivity analysis by excluding the outliers to assess the impact on the pooled estimate was conducted, and in the sensitivity analysis, all of the leave-one-out estimates were within the confidence interval of the pooled estimate. In addition, a sub-group analysis to explore the source of heterogeneity & outliers was done. If we exclude those studies, the overall pooled **mortality could be compromised**. On the other hand, it could not be representative of the mortality rate of HIV-TB co-infected patients in Ethiopia. So, by considering this, we include those studies found in the country and we did the aforementioned analysis.

**Concern 5:** On the subgroup analysis, Tigray region has the highest mortality 31.86% (95% CI 8.83-54.88). The abstract does not give the region with the lowest mortality and Figure 4 forest plot of subgroup analysis by region is not readable nor is the click download for Figure 4 readable so I cannot comment on this. The CI spread of 8.83 – 54.88 for Tigray indicates that this region must have a mixture of very high and very low prevalences. The conclusion is that Ethiopia does not have a consistent monitoring, recording, treatment, and health system, and an overall 18.35% prevalence is misleading because of the extreme outliers and imbalance in the regions.

**Authors’ response:** Thank you for your concern. We have accepted and corrected the revised manuscript and tried to show a visible forest plot. As you have said there are outliers, particularly in Tigray region, and Ethiopia as low-income countries don’t have consistent monitoring and health systems for HIV-TB co-infected patients. Our study tried to show this finding (problem) for the concerned body in the country. So, to tackle this public health problem we accept this finding with its limitations.

**Concern 6:** Hazard ratios: the abstract should indicate that hazard ratios have been used and the various ratios for predictors like HR, for example: In the AHR region poor adherence to ART should indicate HR 2.50(95% CI: 1.28-4.91) also for the other HRs.

**Authors response:** Thank you dear reviewer for your suggestions. We have accepted and corrected the revised manuscript.

**Concern 7:** Hazard ratios are computed in the underlying papers and are the ratios between treatment curves (HIV/TB) and curves for corresponding curves for controls (non-HIV/TB). A problem is that the curves in the underlying papers have different time periods, and this may affect comparability. Hazard ratio curves are often linearized by using log HR or log RR. The various graphs for predictors use The DL method and show the overall DL heterogeneity measure but not the component measures (see DerSimonian and Laird approach (DL) comment above).

**Authors response:** Dear reviewer thank you for your valuable concern. As you have said the difference in time period in the underlying paper affects the comparability. To enhance comparability and reduce bias we have linearized the hazard ratio using the natural logarithm of hazard ratio (HR) then we calculate the standard error of log hazard. We use the log hazard and the standard error for meta-analysis. Finally, the pooled log hazard ratio was back-transformed to the original scale by taking the exponent of the pooled log Hazard ratio.

**Concern 8:** MeSH has been used to search and select papers on the basis of key words and phrases. Only 37 out of 886 studies qualified. There is no test that the key words selected satisfy the relationship between the dependent variable mortality and the independent variables like anaemia, poor ART adherence and lack of co-trimoxazole therapy. Independent variables such as low CD4 count, poor nutritional status, male sex, female sex worker, early initiation of ART for TB patients in various papers have not been included in this paper and have not been included in the MeSH search.

**Authors response:** Thank you dear reviewer for your concern. Our objective is mortality and **predictors** among HIV-TB co-infected patients. Variables like anemia, poor adherence, lack of co-trimoxazole therapy, low CD4 count, poor nutritional status, male sex, female sex worker, and early initiation of ART……… are all predictors, and those primary studies reported these variables as predictors. When we use the key terms predictor, associated factor, risk factor, and determinant in our search it refers to those variables. We tried to use MeSH terms for the main keywords but, we didn’t get the MeSH term except the first key term. We have used comprehensive searching by combining the Free text words with the MeSH term and related terms. After searching those studies, we extracted all predictors and did the analysis. Finally, out of 20 predictors, four predictors were significantly associated with the outcome variables (Mortality). We have stated it in the manuscript. To not miss studies that report the mortality and predictors among HIV-TB co-infected patients, we have used different keywords as shown in the supplementary file.

**Concern 9:** The same independent variables have been applied across categories but would vary between children and adults. A logistic regression should be done to ensure that the independent variables demarcate mortality and non-mortality and that these independent variables are included in the MeSH search and model.

The logistic regression has the form P= (e^(a +bx))/(1+e^(a+bx)); P takes on binary values (1= mortality; 0= no-mortality) and b is the vector of independent variables that take continuous values.

**Authors response:** Thank you for your concern. The primary studies in children and adult have similar categories, due to this we applied the same independent variables. The primary studies that report Predictors are cohorts either retrospective or prospective (But studies that report the proportion of mortality are either cross-sectional or cohort). So, the primary studies are categorizing independent variables into mortality and non-mortality and they use survival analysis. Therefore, in my view, logistic regression is not necessary. In addition, by considering the difference between Pediatrics and Adult we were trying to stratify the analysis of children and adult groups. However, the main problem we face is that almost all of the predictors in either of the groups have either one or two studies that report the association between the required predictor and the outcome variables, and analyzing one or two studies is impossible.

**Concern 10:** Background gives WHO mortality rates, rates for Lesotho, Zambia, children, African region, and South East Asia. These would most likely have different predictors so they are not strictly comparable.

**Authors’ response:** Thank you for your concern. But all of these ideas were narrated to show the magnitude and severity of the problem from global to local context in children and adults because our study includes all population groups.

**Concern 11:** For clarity of presentation they should be shown in a table.

**Authors response:** Thank you dear reviewer for your golden suggestion. We tried to present the invisible forest plot by tables.

**Concern 12:** Spelling and grammar: change ‘systemic’ to ‘systematic’, ‘Anemia’ to ‘anaemia’

**Authors response:** Thank you dear review for your concern. We have accepted and corrected the revised manuscript.

**N.B: In this revised manuscript we analyzed the data using STATA 18 and we used the random effects model with the Restricted Maximum Likelihood (REML) method, rather than the previous Dersimonian Liard method. Due to this, there are some decimal point changes in the revised manuscript.**

**Dear reviewer, thank you for your golden concerns. All of your concerns were constructive and they improved our manuscript for publication. If something is unclear/wrong, please let me know again!**

**Reviewers 2 concerns and Authors response.**

**Concern 1:** There are no page and line numbers used in the documents –the authors did not follow PLOS instructions and makes it difficult to refer.

**Authors Response:** Thank you dear reviewer for your suggestion. We have accepted and modified in the revised manuscript.

Concern 2: According to the United States National Institutes of Health, the United Nations and its Children’s Fund (UNICEF), and European Union pediatrics are defined as persons younger than 18 years. Which guidelines did the authors use to categorize pediatrics up to 15 years?

**Authors response:** Thank you for your concern. As you have said Pediatrics age ranges from birth to 18 years. But almost in all settings of Ethiopia in the ART clinics, a person under 15 years of age is treated as a child because the drug dosage differs from those of adults. On the other hand, a person 15-18 years is treated as an adult and their dosage is the same to adults. Accordingly, authors in the primary study consider pediatric under 15 years and for those above 15 years, they consider as adult. Therefore, in this meta-analysis, we consider pediatrics under 15 years.

**Concern 3:** Table 1 years must be corrected for ID 9 and 11.

**Authors response:** Thank you dear reviewer for your concern. We have accepted and corrected in revised version of the manuscript.

**Concern 4:** Reference the software used to analyse. E.g. Endnote version 9.

**Authors response:** Thank you dear reviewer for your concern. We have corrected in the revised manuscript.

**Concern 5:** The authors need to be consistent when referring to HIV-Tb coinfection/ TB-HIV co-**infection** / HIV/TB co-infection.

**Authors response:** Thank you dear reviewer for your suggestion. We have accepted and corrected in the revised manuscript**.**

**Concern 6:** P-value should be written consistently throughout that manuscript with a symbol= or <.

**Authors response:** Dear reviewer thank you for your suggestion. We have accepted and modified in the revised manuscript.

**Concern 7:** All the abbreviations must be written in full before abbreviation.

**Authors response:** Thank you dear reviewer for your suggestion. We have accepted and corrected in the revised manuscript.

**Concern 8:** Page 13 The authors referenced 6 studies but mentioned that five studies were included to examine the association between anemia and mortality.

**Authors response:** Thank you for your suggestion. We have corrected it in the revised manuscript.

**Concern 9:** Page 14 last sentence under “poor adherence” should read “For this predictor publication was not performed due to small number of studies.

**Authors’ response:** Thank you for your golden suggestion. We have corrected it in the revised manuscript.

**Concern 10:** There are some spelling and grammatical errors on the manuscript that need to be corrected. eg, page 14 under extrapulmonary tuberculosis: HIIV-TB should read HIV-TB

**Authors response:** Thank you dear reviewer for your concern. We have accepted and corrected in the revised manuscript.

**Concern 11:** In the discussion when comparing your studies with other studies, indicate the location of the systematic review and meta-analysis you are comparing too. Paragraph 2 and 6.

**Authors response:** Thank you for your suggestion. We have accepted and corrected in the revised version of the manuscript.

**Concern 12:** Indicate when was the test and treat strategy introduced globally and in Ethiopia?

**Authors response:** Dear reviewer thank you for your suggestion. We have accepted and corrected in the revised version of the manuscript.

**Concerns 13:** Discussion: the percentages after the countries should be inside the brackets.

Eg. This finding is in line with studies conducted in Sub-Saharan Africa (SSA) (18.1%) (1), Europe and Latin America (19%) (63), and systematic review and meta-analysis (15%) (64). However, this finding was lower than a study conducted in Eastern Europe (29%) (63), Brazil (27.4%) (65), and a systematic review and meta-analysis (33.56%)

**Authors response:** Thank you for your suggestions. We have corrected in the revised manuscript.

**Concern 14:** Discussion, paragraph 3 “this discrepancy may be related to limited access to appropriate therapy, ……...”. This statement is contrary to the above where the author mentioned that “the other possible justification is the study period, those studies were incorporated studies conducted before the commencement of the test and treat strategy, but in the present study, most of the included studies were conducted after test and treat all strategies which is a proven strategy in reducing mortality.

**Authors response**: Thank you dear for your suggestion. In paragraph two, we want to refer to “our finding is lower than a study conducted in Eastern Europe 29%, Brazil 27.4%, and a global systematic review and meta-analysis (33.56%) (66). The main reason for this discrepancy is that those studies in Eastern Europe, Brazil, and the other global systematic reviews and meta-analyses included studies conducted before WHO launched the test and treat-all strategy, but in the present study, most of the incorporated studies were conducted after test and treat all strategy, as we know the test and treat all strategy reduces the mortality and the comorbidity level in HIV infected individuals, notably among TB infected patients.

**But, in paragraph 3** we want to refer, in Ethiopia, the HIV-TB infected individuals have limited or poor access to ART/Anti-TB, delayed diagnosis, even the services are fee-free, because there is poor awareness and transportation problems, all this increases the mortality rate in Ethiopia. But, HIV-TB-infected individuals in Western Europe, Latin America United Kingdom, Caribbean, Central and South America, Brazil, and Central and West Africa, may have good awareness, good availability, and accessibility of ART/anti-TB services because their income level and literacy status is better than Ethiopia. This is what we hypothesize.

**N.B: In this revised manuscript we analyzed the data using STATA 18 and we used the random effects model with the Restricted Maximum Likelihood (REML) method, rather than the previous Dersimonian Liard method. Due to this, there are some decimal point changes in the revised manuscript.**

**Dear reviewer, thank you for your valuable concerns, if something is unclear/wrong, please let me know again!**

**We author would like to thank the Editor and the reviewers for their constructive concerns.**
