# Supplementary material for: Mortality and predictors among HIV-TB co-infected patients in Ethiopia: A systematic review and meta-analysis
Source: PLoS One. 2025 Jan 6;20(1):e0317048. doi: 10.1371/journal.pone.0317048 (PMC11703055; doi:10.1371/journal.pone.0317048)
Supplement: Attachment — Submitted filename: Response to Editor and Reviewers.docx [file pone.0317048.s008.docx]

**Authors' response to the Editor, and Reviewers' concern**

**Title: Mortality and predictors among HIV/TB co-infected patients in Ethiopia: A Systematic Review and Meta-analysis.**

**Manuscript ID: PONE-D-24-06919.**

**To: PLOS ONE JOURNAL**

**Date: September 26, 2024.**

**Subject: Revision of the manuscript.**

Thank you, very much dear editor and reviewers, for your valuable and constructive suggestions and comments. We found that the comments are very helpful and constructive to further improve the manuscript. This is our point-by-point response of authors to the editor and reviewers’ suggestions and concerns about the manuscript entitled “Mortality and predictors among HIV/TB co-infected patients in Ethiopia: A Systematic Review and Meta-analysis” which has a manuscript ID of “PONE-D-24-06919” given by the journal. It is known that the manuscript has been reviewed by reviewers and sent back to the authors for revision and resubmission. As authors of this manuscript, the comments and concerns raised by the reviewers and editor are highly important enabling us to improve the quality and plausibility of the manuscript. To do so, we have addressed all of the reviewer's concerns point by point. Therefore, we are very pleased to resubmit the revised version of the manuscript for further revision process and facilitation of its publication on PLOS ONE.

We look forward to hearing from you at your earliest convenience.

With best regards.

Corresponding Author

Wubet Tazeb Wondie	E-mail: wubettazeb27@gmail.com.

On behalf of Co-authors.

**Point-by-point Response Letter**

Dear editorial office of PLOS ONE we have presented our point-by-point response in a way that the reviewers' concern is depicted first, and the authors' response has been given immediately next to it.

**Reviewer 1 concerns and Authors’ response.**

**Concern 1: Line** 31 systematic.

**Authors response:** Dear esteemed reviewer, we thank you for your valuable suggestion. We have accepted and corrected the revised manuscript**.**

**Concern 2:** Line Cochrane Q and I2 test statistics.

**Authors response:** Dear reviewer, thank you for your suggestion. We have accepted and corrected the revised manuscript.

**Concern 3:** Line 42 A total of 886 studies were identified, using database searches and keywords. Of these, 37 met the criteria for inclusion. (omit “and were included in this study”).

**Authors response:** Dear reviewer, thank you for your suggestion. We have accepted and corrected the revised manuscript.

**Concern 4:** 44-49 The APA style for hazard ratios is (HR=2.02, 95% CI [1.7, 2.5])

**Authors response:** Dear reviewer, we thank you for your valuable suggestion. We have accepted and corrected the revised manuscript**.**

**Concern 5:** Line 44-49 APA style for p-values is p<.001 or p≤.05 not p=.00 i.e. state a limit. I know STATA 18 gives the form p=0.00 in the forest plots, but in a formal report, use the APA style (p=0.00 becomes p<.01).

**Authors response:** Dear esteemed reviewer, thanks for your concern. We have accepted and corrected the revised manuscript.

**Concern 6:** Line 50 Nearly one-fifth? How much? present the value, for example, 19.6%.

**Author response:** Dear reviewer. We thank you for your valuable suggestion**.** In response to your suggestion, we have accepted and corrected the revised manuscript.

**Concern 7:** The introduction needs to be rewritten, please refer this to an experienced editor. What is the African region (line 69) and South-East Asian region, line (69). Line 70, what is WHO Africa region? These are vague.

**Authors response:** Dear esteemed reviewer, we thank you for your valuable concern and suggestion. We have accepted and rewritten the introduction. We kindly request you to review the revised manuscript.

**Concern 8:** Line 73 high-HIV-TB burden countries like Ethiopia. This is vague.

**Authors response:** Dear esteemed reviewer, we thank you for your important concern. We have accepted and corrected our write-up.

**Concern 9:** Line 74 $78, is this US dollars?

**Authors response:** Dear reviewer, for your concern. Yes, it is based on US dollars.

**Concern 10:** Line 75 Several studies names 7 factors. Are these the only ones? Did all the studies deal with the same 7 factors? (what about NCDs, cancers?). (No in fact 20 were considered in line 265 – you need to disclose this fact here).

**Authors response:** Dear esteemed reviewer, we thank you for your valuable suggestion. We have accepted your suggestion and added the rest predictors in the revised manuscript.

**Concern 11:** Line 85 46% were getting ART. Does this apply to Ethiopia?

**Authors response:** Dear reviewer, we thank you for your valuable concern. This figure is a global figure and it may not fully represent Ethiopia so we removed it.

**Concern 12: Line 89 Different corners? State "Ethiopia**

**Authors response:** Thank you dear esteemed reviewer for your concern. We have accepted and corrected in revised version of the manuscript.

**Concern 13: Line 96 Systematic.**

**Author response:** Dear **t**hank you for your concern. We have accepted and corrected it.

**Concern 14:** Line 123 Capitalisation of headings is inconsistent, for example, Eligibility Criteria, capitalises both words. Elsewhere only the first is capitalised.

**Authors response**: Dear reviewer, we thank you for your suggestion for clarity of our manuscript. We have accepted your suggestions and corrected in the revised manuscript.

**Concern 15:** Line 124 Systematic

**Authors' Response:** We have accepted and corrected in the revised manuscript.

**Concern 16:** Line 127 Too many hyphens.

**Author response: Dear reviewer,** thank you for your suggestion we have accepted and made corrections in the revised manuscript.

**Concern 17:** Line 128 English (drop language).

**Authors response:** Dear honored reviewer, thank you for your valuable suggestion. We have accepted and corrected the revised manuscript.

**Concern 18:** Line 130 P: HIV-TB etc.

**Authors response:** Dear reviewer, we thank you for your suggestions. We have accepted your suggestion and corrected it in the revised manuscript.

**Concern 19:** Line 143 Software eliminated duplication of studies.

**Authors response:** Dear honored reviewer, we thank you for your suggestion. We have accepted and corrected it in the revised manuscript.

**Concern 20:** Line 144 Briggs (JBI) critical appraisal tools.

**Authors response:** Dear esteemed reviewer, thank you for your suggestion. We have accepted and corrected it in the revised manuscript.

**Concern 21:** Line 153 the pooled hazard ratios (PHR) with 95% confidence intervals.

**Authors response:** Dear reviewer, we thank you for your suggestion. We have accepted your suggestion and corrected it in the revised manuscript.

**CONCERN 22:** Line 158 A restricted maximum likelihood (REML) means a restricted conclusion. How does this affect your study?

**Authors Response:** Dear esteemed reviewer, we express our gratitude for your concrete and golden concern. The most widely used method in the random effects model is the Der Simonian-Liard method. However, when the effect sizes are not normally distributed the restricted maximum likelihood (REML) method is more robust in providing estimates that are less influenced by outliers. A restricted maximum likelihood (REML) method is particularly effective for estimating study variance (heterogeneity) because it focuses on the residual after accounting for the fixed effects, which leads to better estimates. On the other hand, the Der Simonian-Liard method assumes the effect sizes are normally distributed, which can lead to biased estimates when the assumption is violated.

**In General, due to the non-normal distribution of our data, we prefer the restricted maximum likelihood (REML), to account for the heterogeneity and get a reliable estimate. The REML doesn’t give a restricted conclusion rather it gives an unbiased estimate in case of non-normal distribution.**

**Concern 23:** Line 166 p≤.05 APA style.

**Authors response:** Dear reviewer, we thank you for your suggestion. We have accepted and corrected it in the revised manuscript.

**Concern 24:** Line 168 PHR already defined in line 153.

**Author response:** Dear reviewer, we thank you for your suggestion. We have accepted your suggestions and deleted the repeated sentence.

**Concern 26:** 194 Table 1. Style is inconsistent. Mortality % column studies should all use the same number of decimal points.

**Authors response:** Dear reviewer, we thank you for your valuable concern for the clarity of our study. We have accepted your suggestion and corrected it in the revised manuscript.

**Concern 27:** Line 194 ID 6 remove “all over”. Stating “Ethiopia” is sufficient.

**Authors response:** Dear reviewer, thank you for your suggestion. We have corrected it in the revised manuscript.

**Concern 28:** Lines 194 Some authors have initials, others do not.

**Authors response:** Dear reviewer, we thank you for your suggestions. We have accepted and corrected it.

**Concern 29:** Line 194 ID 11 What is 2012S?

**Authors response:** Dear respected reviewer, we thank you for your suggestion. We have accepted and corrected it in the revised manuscript.

**Concern 30:** Line 194 Line up follow-up period dates.

**Authors response:** Thank you dear esteemed reviewer for your suggestions. We have accepted and corrected it in the revised manuscript.

**Concern 31:** Line 194 IDs 14, 17, 18, 19, 20, 22, 23, 24 … 37 have commas after name, others do not.

**Authors response:** Dear respected reviewer, we thank you for your suggestion to the clarity of our manuscript. We have accepted your suggestion and corrected it in the revised manuscript.

**Concern 32: (S3). ( ?**

**Authors response:** Dear reviewer, we thank you for your suggestions. We have accepted and corrected it in the revised manuscript.

**Concern 33:** Line 200 Pooled proportion of mortality

**Authors response:** Dear reviewer, thank you for your comment. We have accepted your comment and corrected it in the revised manuscript.

**Concern 34:** 202 Death rates the of 4.4% …. to 80.45%.

**Authors response:** Thank you dear esteemed reviewer for your suggestion. We have accepted and corrected it in the revised manuscript.

**Concern 35:** Lines 202 to 205 A forest plot weights studies by relative sample size, which is not the same thing as prevalence, which is weighted on relative population size. If the 80.45% applies to 90% of the population of Ethiopia it has a different effect than if it applied to (say) only 10% of the overall population**.**

**Authors Response:** Dear esteemed reviewer, thank you for raising this golden concern. As you have said forest plot weight studies by sample size, inverse variance method, quality of studies……... rather than population size. During analysis, we consider these points, and in the present study, studies were weighted by their sample size. In this regard, we are ready to accept any of your recommendation’s dear reviewer.

**Concern 36:** In Table 3 on page 13, the 18.42% mortality for Ethiopia is weighted on sample sizes of included studies. Table 3 shows number of studies, event, and sample sizes. The discussion of Table 3 should disclose how weighting was applied, for example, did weighting include information in all three columns, or was it based, as I suspect, on sample size only. The 18.42% must be described as weighted on sample sizes (if this is the case), and that this is used as a best proxy for prevalence. Prevalence takes relative population sizes of regions into account. If you do not have these figures, you must qualify your statistic, otherwise your study is invalid.

**Authors Response:** Dear reviewer, we thank you for your golden suggestion. We apologize for any confusion caused by our presentation. The weighting in Table 3 is based on the sample size. Just We showed this table to depict the proportion of mortality in each region in addition to the forest plot (Figure 5). We think that it creates confusion for the reader. So, we have removed those columns and presented the rest statistics. We hope the figure tells more. We are ready to accept any of your comments in this regard.

**Concern 37:** (203 I know the Tigray region was weighted in the forest plot to get a weighted overall effect size and 95% CI. How have you done your weighting of Tigray and other regions to get to your 18.42% overall mortality for Ethiopia? (See comment above).

**Authors response:** Dear reviewer, thank you for your valuable concern. To weight studies in the Tigray region, we use equal weights because there is a similar sample size to the other regions**.** We are ready to accept any of your suggestions in this regard.

**Concern 38:** Lines 205-208 For p-value and CI disclosure, see APA style in lines 44-49.

**Authors response:** Thank you dear esteemed reviewer for your suggestions. We have accepted and corrected it in the revised manuscript.

**Concern 39:** Lines 213-216 See APA style for disclosure.

**Authors response:** Dear reviewer, thank you for your valuable suggestion for the clarity of our manuscript. We have accepted and corrected the revised manuscript.

**Concern 40:** Lines 214 Fig 3 shows many studies outside the 95% CI lines, so the trim and fill exercise is unsuccessful. You need to discuss this or acknowledge the problem and state how you have overcome this. Your conclusion on the 18.42% must be qualified as having been based on a heterogeneous spread of papers. It may help to justify the 18.42% by basing it on population sizes and minimizing the effect of the Tigray region’s outlier effect by stating, for example, that Tigray forms only 5% of the Ethiopian population. How have other countries arrived at their prevalence rates?

**Authors Response:** Thank you, dear esteemed reviewer, for your golden concern and critical view. As you have said in figure3, some studies were outside the 95% CI, because trim and fill and fill analysis is unsuccessful in case of substantial heterogeneity. Due to this, we acknowledge the presence of substantial heterogeneity as a limitation, and the pooled 18.42% mortality is based on heterogeneity spread. In this regard, we are ready to accept any of your potential recommendations.

**Concern 41:** Line 216 (Table 2). ( ?

**Authors' Response:** Dear reviewer, thank you for your suggestions. We have accepted and corrected it in the revised manuscript.

**Concern 42:** Line 219 Table 2 does not state a conclusion and is meaningless in terms of the problem in line 214.

**Authors Response:** Dear reviewer, we sincerely appreciate your critical concern. As you have said it doesn’t support the conclusion. Just we presented it to show the presence of publication bias and the trim and fill analysis. After considering this, it might create confusion for the readers so we have removed the table and the figure has been put as the previous one.

**Concern 43:** Line 227 The sensitivity analysis to resolve the outliers in the Galbraith plot is unsuccessful because many studies lie outside the 95% CI bands. You need to explain why this is the case, if this is important, and how you have discounted this in your conclusion.

**Authors response**: Dear reviewer, thank you for your critical concern. In the sensitivity analysis, the **single estimate in each omitted study lies within the overall 95% CI (95% CI;14.27-22.57).** None of the single estimates (effect size lies) in the omitted study lies out the 95% CI. Take a look at the single estimate of the omitted study in the sensitivity analysis and the overall estimate in the forest plot.

**Concern 44:** Lines 233 to 238 See APA disclosure of p-values and confidence intervals.

**Author response:** Dear, we thank you for your concern. We have accepted and corrected in the revised manuscript.

**Concern 45**: Lines 242-243 See the forest plot presentation of effect size and 95% CI used by STATA 18.

**Authors response:** Dear thank you for your concern. We have seen it. To some extent, it is not visible. We have shown a table to support this figure (Table 3).

**Concern 46:** Line 242-243 The 18.42[14.27, 22.57] for Ethiopia is based on sample size. Prevalence needs to be weighted by relative population size (see discussions above).

**Authors response:** Dear reviewer, we sincerely appreciate your critical comment. As we have mentioned above, in our study weighting is based on sample size. We hope the forest plots will tell more.

**Concern 47:** Lines 248 Remove furthermore.

**Authors response:** Dear esteemed reviewer, thank you for your suggestions. We have accepted and corrected it.

**Concern 48:** Line 250 Remove accordingly.

**Authors response:** Thank you dear reviewer for your concern, we have accepted and corrected it.

**Concern 49:** 251-254 For effect size and CI, use STATA 18 forest plot form, for formal disclosure p-value use the APA form.

**Authors response:** Dear reviewer thank you for your concern. We have accepted and corrected in the revised manuscript.

**Concern 50:** Line 265 20 predictors assessed. These are not mentioned in your abstract on page 2. Only the 7 significant ones are shown in lines 50-53. The abstract on page 2 is incomplete. You are concluding on something that has not been included in your opening statements of aims.

**Authors response:** Dear honored reviewer, we thank you for your valuable concern. As we stated in the document, we assessed the significant association of 20 predictors with the outcome variable independently, using the random effects model. We have accepted your concern and have stated that we have tested the 20 predictors and show only the four statistically significant variables in the forest plot.

**Concern 51:** Lines 265-272 You cannot use a forest plot to assess which predictors are significant. A forest plot is a meta-analysis of a particular property in a set of publications. It is not a direct statistical model to assess individual significance in a set of multiple variables which are likely to be interdependent. This can only be done by regression models, ANOVA models or other multivariable statistical models. You need to design a matrix of dependent and independent variables and a regression analysis (for example a multiple linear regression, or preferably a logistic regression). You are attempting to gauge significance of individual variables by subgroup forest plots. The more subgroup analyses you do, the smaller is the sample size of data which you are testing, which may affect significance achieved, if sample sizes are too small. The testing of 20 variables as independent predictors is incorrect unless you can demonstrate they are independent. A correct test is to include these into a matrix with a column for the dependent variable (HIV1-TB) and to determine model fit (root mean square values), F statistics and p values and to remove variables with p values >.05.

**Authors response:** Dear esteemed reviewer, we sincerely appreciate your critical comment. As you have said, in a forest plot we can’t assess individual significance in a set of multiple variables**.** In this regard, in the forest plot, we assessed the statistical significance of each variable **independently using the random effect model, and we attached the significant variables in the manuscript. The statistical significance of each variable is assessed independently. Our study is meta-analysis no more multivariable analysis like primary study. (i.e. one variable is tested in one forest plot).** In this regard, we are ready to accept any of your recommendations!

**Concern 52:** Lines 265-272 You cannot assess predictors singly. There will be some collinearity (dependence between one or more predictors) See comment above.

**Authors response:** Dear reviewer, we thank you for your concern. As mentioned above, the statistical significance of each predictor is assessed independently. There could be no collinearity between predictors because the statistical significance test is assessed independently (separately). i.e. one variable is tested in one forest plot.

**Concern 53:** Lines 325-336 comparisons between countries. You need to justify why these are comparable. Were they done on the same basis as your analysis? Are they claiming prevalence or are they based on your sample weighting basis?

**Authors response:** Dear reviewer, thank you for your valuable concerns. The studies we compared from lines 325-336; some of them are systematic reviews and meta-analysis of observational studies, and the other are observational studies conducted on large populations that include more than 10,000 samples. Hence, some of the studies have the same analysis as our study, and some of them are national studies that include large population groups with the same (Population, Exposure, Comparison, and Outcome (PECO)) as the present study. Due to the same analysis method and similarities of PECO, we compared those studies to our finding.

**Concern 54:** Lines 337 heterogeneity indicates to what extent studies do not come from a common source or population. The Tigray region is an outlier, and there are relatively few studies to do a test of more than a few predictors. This is a major issue, and a hazard ratio may not be significant because of this.

**Authors Response:** Dear esteemed reviewer thanks for your critical insight and suggestion. Line 337 showed the subgroup analysis about the proportion of mortality and Tigray region has a high proportion of mortality. The reasons for this heterogeneity are stated in the main manuscript. Like poor adherence to treatments of HIV/AIDS and TB in Tigray region, poor treatment outcomes of TB patients and poor awareness in Tigray region, and so on. To pool the hazard ratio of each predictor we collect hazard ratios from each study with respect to each predictor and we made our analysis in this way. In this regard, we are ready to accept any of your recommendations.

**Concern 55:** Line 337 If there is interdependence between predictors, it may affect the significance of the hazard ratios you are computing as isolated predictors.

**Authors response:** Dear honored reviewer, we thank you for your valuable concern. There was no interdependence between predictors in our analysis and it doesn’t affect the significance of the hazard ratio. We collected the hazard ratio of each predictor independently and computed the pooled effect size independently for each predictor.

**Concern 56:** Line 387 Strengths and limitations. This indicates major limitations, for example, high heterogeneity, unavailability of studies and inconsistent measurements. This means that the result of 18.42% proportion of mortality is in doubt and that your study has failed. This is a significant problem. You can circumvent this problem by qualifying your figure as based on a best proxy and justifying it by referring to other countries who have used the same basis.

**Authors response:** Dear reviewer, we sincerely thank you for your valuable concern. As you have said the stated limitations are significant problems. This study includes all parts of Ethiopia because there are studies conducted in each region and also there are studies conducted across all parts of Ethiopia, we have removed this limitation. But for the rest limitations (heterogeneity, and inconsistent measurements of nutritional status), we have acknowledged them as a limitation. The articles we used for discussion have the same basis.

**Dear reviewer, we thank you for your valuable concerns. All of your concerns were constructive and they helped us to improve our manuscript for publication. If something is unclear/wrong, please let me know again!**

**Reviewer #2:** The authors addressed all my comments to my satisfaction and the manuscripts is written in a scientific way.

**Authors Response:** Dear esteemed reviewer, we thank you for your kind words, and consideration

**We author would like to thank the Editor and the reviewers for their constructive concerns.**
