# Supplementary material for: Mortality and predictors among HIV-TB co-infected patients in Ethiopia: A systematic review and meta-analysis
Source: PLoS One. 2025 Jan 6;20(1):e0317048. doi: 10.1371/journal.pone.0317048 (PMC11703055; doi:10.1371/journal.pone.0317048)
Supplement: Attachment — Submitted filename: Response to Editor and Reviewer R3.docx [file pone.0317048.s009.docx]

**Authors' response to the Editor, and Reviewers' concern**

**Title: Mortality and predictors among HIV/TB co-infected patients in Ethiopia: A Systematic Review and Meta-analysis.**

**Manuscript ID: PONE-D-24-06919.**

**To: PLOS ONE JOURNAL**

**Date: November,4, 2024.**

**Subject: Revision of the manuscript.**

Thank you, very much dear editor and reviewers, for your valuable and constructive suggestions and comments. We found that the comments are very helpful and constructive to further improve the manuscript. This is our point-by-point response of authors to the editor and reviewers’ suggestions and concerns about the manuscript entitled “Mortality and predictors among HIV/TB co-infected patients in Ethiopia: A Systematic Review and Meta-analysis” which has a manuscript ID of “PONE-D-24-06919” given by the journal. It is known that the manuscript has been reviewed by reviewers and sent back to the authors for revision and resubmission. As authors of this manuscript, the comments and concerns raised by the reviewers and editor are highly important enabling us to improve the quality and plausibility of the manuscript. To do so, we have addressed all of the reviewer's concerns point by point. Therefore, we are very pleased to resubmit the revised version of the manuscript for further revision process and facilitation of its publication on PLOS ONE.

We look forward to hearing from you at your earliest convenience.

With best regards.

Corresponding Author

Wubet Tazeb Wondie	E-mail: wubettazeb27@gmail.com.

On behalf of Co-authors.

**Point-by-Point Response Letter**

Dear editorial office of PLOS ONE we have presented our point-by-point response in a way that the reviewers' concern is depicted first, and the authors' response has been given immediately next to it.

**Reviewer 1 Concerns and Authors’ Response.**

**Concern 1:** General: reporting of data especially p-values and 95% CLs is inconsistent. STATA shows exact p-values, p=0.00 so references to forest plots must show the exact values. Sometimes you adopt the STATA representation of CIs as in the square brackets in line 246 and sometimes your depiction with round brackets. A p-value gives the probability that a statistic like i^2 is a chance value. If (say) i^2=98.002, which indicates high heterogeneity, p=0.11 is not significant, because it is higher than p=0.05 and we cannot conclude that the i^2 is significant or not (a p-value is usually computed by a chi-square distribution, and a statistic such as i2 does not determine its own p-value).

**Authors response:** Dear esteemed reviewer, we thank you for your valuable concern. We have accepted your suggestion and corrected it in the revised manuscript. For heterogeneity, we used Tau-squared and other statistics as shown in the forest plot in addition to the I2 value. Thanks again.

**Concern 2:** Lines 45-46 the lowest pooled mortality was reported for two general studies in Ethiopia 11.95 (95% CI; 4.19-19.00).

**Authors' response:** Dear respected reviewer, we thank you for your concern, we have accepted and corrected the revised manuscript.

**Concern 3:** Line 51 … 18.42% (95% CI; 14.27-22.57), i2=98.02%. The lowest individual study mortality was 4.40 (95%CI; 1.21-7.50) and the highest, 80.48 (95%CI; 75.12-85.84), which indicated significant inter study heterogeneity. This was confirmed by the funnel and Galbraith plots, where 22 out of the 37 studies lay outside the central pseudo-95% region. The tau-squared value of 160.41 indicated there was significant within study variability. In the subgroup analysis…

**Authors response:** Dear respected reviewer, we thank you for your valuable concern. As you have said there was inter-study heterogeneity, and 22 out of the 37 studies lay outside the central pseudo-95% region. Accordingly, we have stated briefly this inter-study heterogeneity in the subgroup section from lines 234-258. Dear reviewer, in this regard we are ready to accept any of your recommendations.

**Concern 4:** Line 65 …which accounts for 30%...

**Authors response:** Dear reviewer, thank you for your concern, we have accepted and corrected the revised manuscript.

**Concern 5:** Line 164-167 (this is a conceptual section, not a results section). …to assess the presence of a small study effect, an Egger’s test was done. The trim and fill funnel plot analysis was confirmed by a Galbraith plot, and the studies lying inside and outside the central 95% pseudo-CI region was agreed to the distributional features in the pooled mortality forest plot in Fig 2.

**Authors response:** Dear respected reviewer we thank you for your important suggestions. We have accepted your suggestions and corrections are taken.

**Concern 6:** Line 205-207 rewrite this (note that p values in forest plots are exact in STATA forest plots).

**Authors response:** Dear esteemed reviewer, thank you for your concern. We have accepted and corrected it in the revised manuscript.

**Concern 7:** Line 205-207 significant heterogeneity was observed between studies with a Q=123.16; and i^2 =98.02 and H^2 =50.46; P=0.00. Tau-squared=160.41 indicated significant heterogeneity within studies.

**Authors response:** Dear reviewer thank you for your concern. As you have mentioned, Tau squared and other statistics indicate a significant heterogeneity, and we have narrated it in the revised manuscript.

**Concern 8:** Line 211-226 I suggest a rewrite of these lines as follows –

Line 211-215 An Egger’s test value, P<0.001, indicated a significant publication bias, and highly non-normal distribution. A funnel plot confirmed both the bias and the non-normal distribution, but the non-parametric trim and fill analysis failed to resolve the bias because 22 of the 37 studies and the 3 imputed studies remained outside the pseudo 95% CIs. Standard errors to effect size (mortality) varied between 6.5 standard errors and 0.5 standard errors before and after the three imputed studies. A Galbraith plot confirmed the results of the funnel plot.

**Authors response:** Dear esteemed reviewer, we thank you for your valuable concern. We have accepted your recommendation and corrected it in the revised manuscript.

**Concern 9:** Lines 216 Fig 3: Non-parametric funnel plot with fill and trim analysis

**Authors response:** Dear esteemed reviewer we thank you for valuable suggestions. We have accepted and corrected in the revised manuscript.

**Concern 10:** Line 217 Fig 4: Galbraith plot

**Authors response:** Dear respected reviewer, thank you for your suggestions. We have accepted and corrected it in the revised manuscript.

**Concern 11:** Line 218-226 Handling heterogeneity

Because of the high heterogeneity between and within the 37 studies, a forest plot was prepared using the REML method to show the pooled mortality for the 37 studies (Fig 2). This confirmed the significant heterogeneity of the Egger’s test and the significant bias of the funnel plot, and identified the 22 studies falling outside the 95% pseudo-CI region of the funnel plot. Although the pooled mortality in the forest plot was 18.42% (95% CI; 14.27-22.57), there was a significant range in mortality values for Ifa 2018, 4.40% (95% CI; 1.21-7.59), to Habtamu et.al. 2021, 80.48% (95% CI; 75.12-85.84). In view of the heterogeneity, subgroup and sensitivity analyses were performed by region (Fig 5), and by age (Fig 6).

**Authors response:** Dear respected reviewer, we thank you for your valuable comments. We have accepted and corrected it in the revised manuscript.

**Concern 12:** Line 227 Subgroup analysis, Line 228-239 Some figures in 228 to 236 do not agree with the table in 239. For example, Addis Ababa and Amhara region and SNNPR.

**Authors response:** Dear respected reviewer, thank you for your golden suggestion**.** We have accepted and corrected it in the revised manuscript.

**Concern 13:** Line 233 to 236 Is incorrect. The subgroup analysis is between groups not within groups. Within group heterogeneity is significant (all i^2 are high) at p=0.00, which means that the heterogeneity values are not obtained by chance. The test of group differences, that is, the between group differences shows significant heterogeneity with i^2 = 98.02. However, p=0.30 for group differences, shows that there is a 30% chance that the heterogeneity values are incorrect, which is much higher than the benchmark p=0.05 and so are not significant (once again STATA gives exact p-values in the forest plots).

**Authors response:** Dear esteemed reviewer, we thank you for your concern. We have accepted your suggestions and corrected it in the revised manuscript. However, the I^2^= 98.02 is for the overall pooled estimate not for between studies, but the P-value =0.30 is for between studies heterogeneity (test of group differences). Dear reviewer in this regard, we are ready to accept any of your recommendations.

**Concern 14:** Line 239 The table should show a column between Region and Proportion ((95% CI) for study size. For example, Addis Ababa| 3 | 20.36 (1.76-36.97) | 68.22| 97.82| 0.00| and for the rest of the rows. This emphasises that some areas have very low study sizes.

**Authors response:** Dear respected reviewer, we thank you for your valuable concern. We have accepted and corrected it in the revised manuscript.

**Concern 15:** Lines 246-248 Is incorrect. The test for group differences had p=0.11 which is above p=0.05 and is not significant because there is a 11% chance that the high heterogeneity values are incorrect. The within group significant heterogeneity is significant at p=0.00 or p=0.01.

**Author response:** Dear respected reviewer we are grateful for your golden concern, we found these concerns to be helpful. We have accepted and corrected it in the revised manuscript.

**Concern 16:** 256 Add that the weights in Fig 2 are approximately equal and group around 100/37=2.70, which is satisfactory and shows that no study unduly influences the overall weighting.

**Author response:** Dear respected reviewer, we thank you for your insightful suggestions. We have accepted your suggestion and corrected it in the revised manuscript.

**Concern 17:** 258 …in this study, the pooled effect size of the 20 independent predictors was assessed.

**Authors response:** Dear respected reviewer, we thank you for your suggestions. We have accepted your comment and corrected it in the revised manuscript.

**Concern 18:** 297 Incorrect. A p-value is the chance that a test is by chance or incorrect. P=0.244 means one cannot accept the significance or non-significance of the test.

**Author response:** Dear reviewer, thank you for your valuable concern. The Eggers test result showed a P-value of 0.244 which is above the benchmark 0.05, this suggests that there is no statistically significant evidence of asymmetry in the funnel plot. This means that we don’t have strong evidence to conclude that the publication bias is present in this predictor, and we tried to show this in the manuscript. Dear reviewer, in this regard we are ready to accept any of your recommendations.

**Concern 19:** 380-381 our studies had the following limitations: the significant heterogeneity in the pooled proportion of mortality of the 37 studies, remained in the subgroup and sensitivity analysis and within study analysis. The funnel plot and Galbraith plot analysis indicated significant bias which was not resolved in the fill and trim procedure in the case of the funnel plot. Nevertheless, the paper has significant value in establishing benchmark values in the case of a country with significant diversity in its independent predictors.

**Authors response:** Dear reviewer, we thank you for your golden concern, we found that your concern is significantly important for the clarity of our study. We have accepted your suggestions and we take corrections in the revised manuscript.

**Dear reviewer, we thank you for your valuable concerns. All of your concerns were constructive and they helped us to improve our manuscript for publication. If something is unclear/wrong, please let me know again!**

**We author would like to thank the Editor and the reviewer for their constructive concerns.**
