# Supplementary material for: Mortality and predictors among HIV-TB co-infected patients in Ethiopia: A systematic review and meta-analysis
Source: PLoS One. 2025 Jan 6;20(1):e0317048. doi: 10.1371/journal.pone.0317048 (PMC11703055; doi:10.1371/journal.pone.0317048)
Supplement: Attachment — Submitted filename: Response to Editor and Reviewer R4.docx [file pone.0317048.s010.docx]

**Authors' response to the Editor, and Reviewers' concern**

**Title: Mortality and predictors among HIV/TB co-infected patients in Ethiopia: A Systematic Review and Meta-analysis.**

**Manuscript ID: PONE-D-24-06919.**

**To: PLOS ONE JOURNAL**

**Date: December 1, 2024.**

**Subject: Revision of the manuscript.**

Thank you, very much dear editor and reviewer, for your valuable suggestions and comments. We found that the comments are very helpful and constructive to further improve the manuscript. This is our point-by-point response of authors to the editor and reviewers’ suggestions and concerns about the manuscript entitled “Mortality and predictors among HIV/TB co-infected patients in Ethiopia: A Systematic Review and Meta-analysis” which has a manuscript ID of “PONE-D-24-06919” given by the journal. It is known that the manuscript has been reviewed by reviewers and sent back to the authors for revision and resubmission. As authors of this manuscript, the comments and concerns raised by the reviewers and editor are highly important enabling us to improve the quality and plausibility of the manuscript. To do so, we have addressed all of the reviewer's concerns point by point. Therefore, we are very pleased to resubmit the revised version of the manuscript for further revision process and facilitation of its publication on PLOS ONE.

We look forward to hearing from you at your earliest convenience.

With best regards.

Corresponding Author

Wubet Tazeb Wondie	E-mail: wubettazeb27@gmail.com.

On behalf of Co-authors.

**Point-by-Point Response Letter**

Dear editorial office of PLOS ONE we have presented our point-by-point response in a way that the reviewers' concern is depicted first, and the authors' response has been given immediately next to it.

**Editor concern:** Please review your reference list to ensure that it is complete and correct. If you have cited papers that have been retracted, please include the rationale for doing so in the manuscript text, or remove these references and replace them with relevant current references. Any changes to the reference list should be mentioned in the rebuttal letter that accompanies your revised manuscript. If you need to cite a retracted article, indicate the article’s retracted status in the References list and also include a citation and full reference for the retraction notice**.**

**Author response:** Dear respected Editor, we thank you for your golden concern. Based on your recommendation, we have reviewed all of the references, and none of them have been retracted. All references from journals or web pages are available. Thanks again.

**Reviewer 1 Concerns and Authors’ Response.**

**Concern 1:** Line 45, 31.86% (95% CI: 7.69 - 56.03)

**Authors response:** Dear esteemed reviewer, we thank you for your valuable suggestions. We have accepted and corrected the revised manuscript.

**Concern 2: Line,47-48. anaemia (HR = 2.25, 95% CI: 1.5 – 3.07)**

**Authors response:** Dear respected reviewer, thank you for your concern, we have accepted your suggestion and corrected it in the revised manuscript.

**Concern 3:** Line 49, co-trimoxazole preventive therapy (HR = 1.87, 95% CI: 1.28 – 2.73).

**Authors response:** Thank you dear reviewer for your comments. We have accepted and corrected it in the revised manuscript**.**

**Concern 4:** Line 50 (HR = 1.23, 95% CI: 1.01 – 1.51)

**Authors response:** Thank you dear reviewer for your suggestions. We have accepted your suggestions and corrected the revised manuscript.

**Concern 5: Line 66 [6]. (full stop)**

**Authors response:** Dear respected reviewer, thank you for your valuable suggestions. We have accepted your suggestion and corrected it in the revised manuscript.

**Concern 6: Line 107 Systematic**

**Authors' response:** Dear reviewer, we thank you for your suggestion, we have accepted and corrected it.

**Concern 7:** Line 108 Systematic

**Authors response:** Thank you, dear reviewer, for your suggestions we have accepted and corrected it.

**Concern 8:** Line 153 predictors of mortality

**Authors response:** Dear esteemed reviewer, thank you for your comment, we have accepted and corrected in the revised manuscript.

**Concern 9:** Line 236 31.86% (95% CI: 7.69 – 56.03)

**Authors response:** Dear esteemed reviewer, thank you for your comment, we have accepted and corrected the revised manuscript**.**

**Concern 10:** Line 237 20.73% (95% CI: 15.15 – 26.30)

**Authors response:** Authors response: Dear esteemed reviewer, thank you for your comment, we have accepted and corrected the revised manuscript.

**Concern 11:** Line 237-238 20.36% (95% CI: 1.76 – 38.97).

**Authors response:** Dear esteemed reviewer, thank you for your comment, we have accepted and corrected the revised manuscript**.**

**Concern 12:** Line 238 15.19% (95% CI: 10.73 – 19.65) 13.66% (95% CI: 8.98 – 18.35)

**Authors response:** Dear respected reviewer, thank you for your concern, we have accepted and corrected the revised manuscript.

**Concern 13:** Line 239 13.74% (95% CI: 6.86 – 20.62)

**Authors response:** Thank you dear esteemed reviewer for your suggestions, we have accepted and corrected it.

**Concern 14:** Line 240 11.95% (95% CI: 4.91 – 19.00)

**Authors response:** Dear esteemed reviewer, thank you for your comment, we have accepted and corrected it in the revised manuscript.

**Concern 15:** Line 242 (regions)

**Authors response:** Dear reviewer, we thank you for your valuable suggestions, we have accepted and corrected it.

**Concern 16:** Line 251 pediatrics

**Authors response:** Thank you dear esteemed reviewer for your suggestions, we have accepted and corrected the revised manuscript**.**

**Authors' response:** Dear respected reviewer, we thank you for your concern, we have accepted and corrected the revised manuscript.

**Concern 17:** Line 252 20.70% (95% CI: 14.29 – 27.11)

**Authors response:** Dear reviewer, we thank you for your valuable suggestions, we have accepted and corrected it.

**Concern 18:** Line 281 hazard ratio

**Authors response:** Dear esteemed reviewer, thank you for your comment, we have accepted and corrected the revised manuscript.

**Concern 19:** Line 290 hazard ratio

**Authors' response:** Dear reviewer, we thank you for your valuable suggestions, we have accepted and corrected it in the revised manuscript.

**Concern 20: Line 308 hazard ratio**

**Authors response:** Dear respected reviewer, thank you for your valuable comment, we have accepted and corrected it.

**Concern 21: Line 313 co-infected**

**Authors' response:** Dear reviewer, we thank you for your suggestions, we have accepted and corrected it.

**Concern 22: Line 314-315 18.42% (95% CI: 14.27 – 22.57)**

**Authors response:** Dear esteemed reviewer, thank you for your valuable suggestion, we have accepted and corrected it.

**Dear reviewer, we thank you for your valuable concerns and time. All of your concerns were constructive and they helped us to improve our manuscript for publication. If something is unclear/wrong, please let me know again!**

**We author would like to thank the Editor and the reviewer for their constructive concerns.**
